# Atmosphere-Induced Transient Structural Transformations of Pd–Cu and Pt–Cu Alloy Nanocrystals

**DOI:** 10.1021/acs.chemmater.1c02377

**Published:** 2021-11-10

**Authors:** Lea Pasquale, Sharif Najafishirtari, Rosaria Brescia, Alice Scarpellini, Cansunur Demirci, Massimo Colombo, Liberato Manna

**Affiliations:** † Department of Nanochemistry, Istituto Italiano di Tecnologia, Via Morego 30, 16163 Genova, Italy; ‡ Dipartimento di Chimica e Chimica Industriale, Università degli Studi di Genova, Via Dodecaneso 31, 16146 Genova, Italy; § Electron Microscopy Facility, Istituto Italiano di Tecnologia, Via Morego 30 16163, Genova, Italy

## Abstract

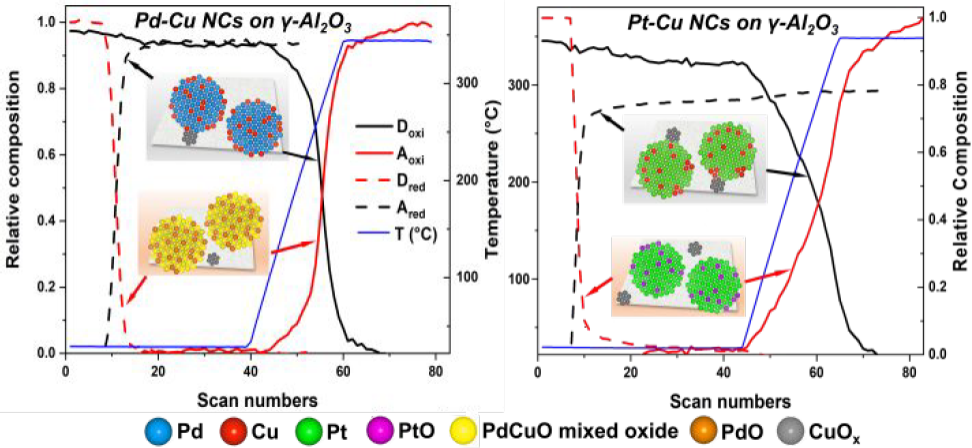

We have investigated the transformations of colloidal Pd–Cu and Pt–Cu bimetallic alloy nanocrystals (NCs) supported on γ-Al_2_O_3_ when exposed to a sequence of oxidizing and then reducing atmospheres, in both cases at high temperature (350 °C). A combination of in situ diffuse reflectance infrared Fourier transform spectroscopy and X-ray absorption spectroscopy was employed to probe the NC surface chemistry and structural/compositional variations in response to the different test conditions. Depending on the type of noble metal in the bimetallic NCs (whether Pd or Pt), different outcomes were observed. The oxidizing treatment on Pd–Cu NCs led to the formation of a PdCuO mixed oxide and PdO along with a minor fraction of CuO_*x*_ species on the support. The same treatment on Pt–Cu NCs caused a complete dealloying between Pt and Cu, forming separate Pt NCs with a minor fraction of PtO NCs and CuO_*x*_ species, the latter finely dispersed on the support. The reducing treatment that followed the oxidizing treatment largely restored the Pd–Cu alloy NCs, although with a residual fraction of CuO_*x*_ species remaining. Similarly, Pt–Cu NCs were partially restored but with a large fraction of CuO_*x*_ species still located on the support. Our results indicate that the noble metal present in the bimetallic Cu-based alloy NCs has a strong influence on the dealloying/migrations/realloying processes occurring under typical heterogeneous catalytic reactions, elucidating the structural/compositional variations of these NCs depending on the atmospheres to which they are exposed.

## Introduction

Heterogeneous catalytic processes involving bimetallic alloy nanocrystals (NCs), including oxidation and reduction reactions, environmental catalysis, electrocatalysis, biomass conversion, and energy storage, have been the subject of several studies.^1−3^ Consequently, significant progress has been made in the synthesis of well-defined alloy NCs by tuning their size, shape, and composition.^4,5^ However, bimetallic alloy NCs can undergo extensive structural transformation, surface segregation and change in their chemical state, structure, and reactivity, upon exposure to reactants and high temperatures.^6−13^ In analogy with monometallic NCs, bimetallic NCs can also suffer from sintering and can be redispersed in certain conditions and depending on gas atmospheres.^14^ Ostwald ripening and particle disintegration induced by reactants are processes that imply different interactions between adsorbate-metal, metal–metal, and metal–support.^15^

Major research efforts have focused on the investigation of complex dynamic processes occurring in bimetallic-based NCs under reaction conditions.^16,17^ Efforts have been made to alloy noble metals (e.g., Au, Pd, Pt) with nonprecious transition metals, such as Cu, also with the aim to alter the electronic properties of the noble metal and to attain cost-affordable, stable NCs with enhanced catalytic performance.^18^ For example, Shan et al.^8^ studied how the surface structure affected the dispersion of metal sites on the surface of shape-controlled Pt–Cu NCs. Their study applied different treatment conditions to vary the NC surface composition and structure, which correspondingly changed their catalytic performance in the CO oxidation reaction. Kalyva et al.^19^ observed a mechanism of Cu leaching out from the Pt–Cu NCs under oxidation–reduction cycles at elevated temperatures. Additionally, it was found that the local composition of alloy NCs can be affected by the presence of the support due to the preferential interaction of one of its elements with the support at the interface. Huang et al.^20^ showed that Pt–Cu alloyed NCs supported on TiO_2_ undergo extensive transformations when exposed to either an oxidizing or a reducing environment, resulting in a surface reconstruction that is different from that of the bulk, highlighting the important role of the support in the dispersion and morphology of the alloyed NCs. Xi et al.^21^ demonstrated that in the case of Pd–Cu NCs supported on WO_2.72_ nanorods, the strong interaction between the NCs and the support stabilized Cu in the NCs in an acidic environment.

Despite the weak interaction of nonreducible oxides such as Al_2_O_3_, SiO_2_, ZrO_2_, and MgO with metals,^22,23^ only a few studies have investigated the mechanism of electronic and/or geometric modifications of noble metal-Cu alloyed NCs in the presence of these supports, generally used to enhance the NCs stability, by controlling the process conditions in selected atmospheres. A recent study, conducted by Le et al.,^24^ demonstrated the role of different supports, such as TiO_2_, SiO_2_, and γ-Al_2_O_3_, in controlling the crystal structure of Pd–Cu NCs, which was reflected in different catalytic activities and selectivities in the hydrogenation of succinic acid.

Previous studies^25−27^ have elucidated the impact of the support on the structural evolution of Au_1–*x*_–Cu_*x*_ colloidal NCs with well-controlled size and composition supported on γ-Al_2_O_3_ and SiO_2_ at high temperature (350 °C) in selected atmospheres. Specifically, it was proven how the type of support impacts the phase segregation between Au and Cu. Through different characterization techniques, it was shown that, under oxidizing conditions, Cu was dealloyed from Au and the formed CuO_*x*_ species had different fates, depending on the support: while they were finely dispersed on alumina and partially migrated away from the Au NCs, the CuO_*x*_ species on silica formed small clusters located in the proximity of the Au NCs, with limited Cu migration on the support. Changing the gas atmosphere to a reducing one restored the Au–Cu alloyed NCs when supported on alumina. This means that changing the gas atmosphere reversed the migration process of Cu. A partial realloying also occurred on the silica-supported Au–Cu NCs, resulting in the formation of Cu-depleted alloy NCs and isolated metallic Cu. For such bimetallic NCs, and under the investigated conditions, Cu was the only element that underwent the dealloying/migration/realloying process as a function of the reacting atmosphere.

**Scheme 1:**
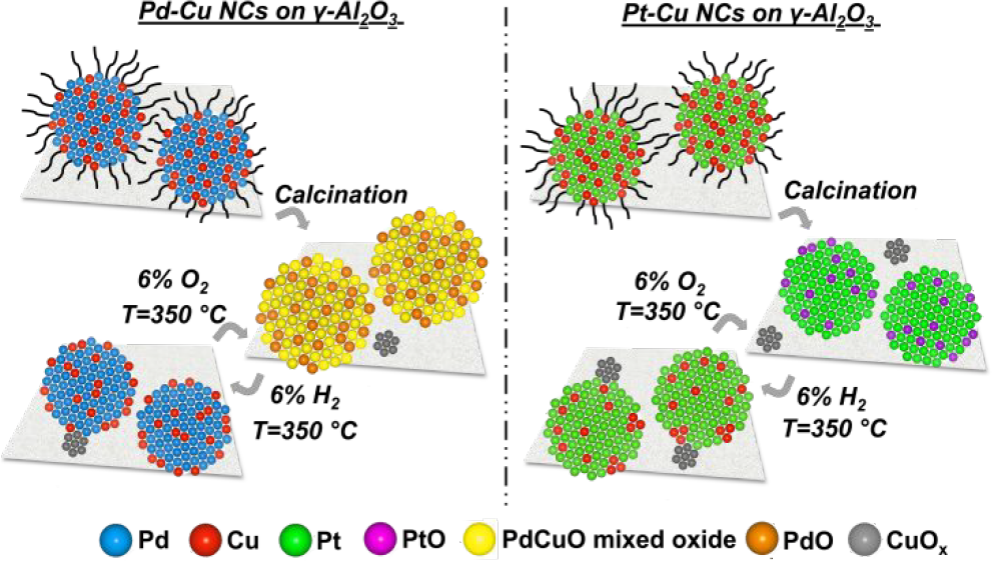
Sketch of the Pd–Cu and Pt–Cu Alloy NC Transformations as a Function of the Reaction Environments

Considering the mobility of Cu on different supports and the role of Au as a reversibility anchor in the alloying/dealloying/migration processes for Au–Cu NCs, we studied here the effect of other two noble metals, i.e., Pd and Pt, in the compositional rearrangements of two families of supported noble metal-Cu alloyed NCs in response to oxidizing and reducing atmospheres at 350 °C, i.e., the same conditions used in the previous studies to effectively remove the ligands from the surface of deposited NCs.^25,26^ Moreover, a detailed characterization was carried out on the initial state of the NCs before exposing them to the reaction mixture for the subsequent catalytic application (CO oxidation reaction, not reported here) along with their transformations upon exposure to the above-mentioned treatments. In this regard, the effects of temperatures beyond 350 °C, i.e., the maximum temperature of the treatment, were not explored. Two types of Cu-based bimetallic alloyed NCs, namely Pd–Cu and Pt–Cu, were synthesized through colloidal synthesis methods, which allowed fine adjustment of size and atomic ratio between the Cu and the noble metal (atomic noble metal: Cu = 50:50). The as-synthesized NCs were deposited on γ-Al_2_O_3_ and calcined in static air to remove the organic ligands present on the NC surface. Their transformations upon oxidative or reductive conditions were studied, applying the same treatment protocols and using the same supports of the previous papers.^25,26^ Among other techniques, in situ diffuse reflectance infrared Fourier transform spectroscopy (DRIFTS) and X-ray absorption spectroscopy (XAS) were specifically used to characterize the evolution of the chemical states and compositions (surface and bulk) after and during the treatments. A varying extent of the alloy/dealloy process, depending on the type of noble metal involved, was observed. During the oxidizing treatment on the Pd–Cu NCs, Cu was mostly retained in the NCs, with the formation of a PdCuO mixed oxide (Scheme 1, left panels), while, in the Pt–Cu NCs case, Cu was found finely dispersed on the alumina support as CuO_*x*_ species, and Pt stayed in the NCs (Scheme 1, right panels). In a reducing atmosphere, an almost complete realloying of the Pd–Cu NCs was found, compared to the partial restoration of the Pt–Cu NCs. These observations highlight the capability of the Pd–Cu system to regenerate its initial structure thanks to the formation of the CuO-Pd interface that promotes the realloying of the Cu.

## Experimental Section

### Colloidal Nanocrystal Synthesis and Treatments

#### Materials

Palladium(II) acetylacetonate (97%), copper(II) acetylacetonate (97%), benzyl ether (99.8%), 1,2-hexadecanediol (90%), oleic acid (OlAc, 90%), oleylamine (OlAm, 70%), platinum(II) acetylacetonate (98%), 1-octadecene (ODE, 90%), borane morpholine complex (MB, 95%), tertbutylamine-borane complex (TBAB, 97%), copper(I) acetate (97%), trioctylamine (98%), and solvents (anhydrous chloroform, anhydrous isopropanol, toluene, and hexane) needed to synthesize the NCs were purchased from Sigma-Aldrich and used as received without further purification. γ-Al_2_O_3_ powder (extrudate from Sigma-Aldrich, crushed and sieved to 90 μm mean size, BET specific surface area 190 m^2^ g^–1^) was purchased from Strem Chemicals.

#### Bimetallic Alloy Nanocrystals

NCs were synthesized by employing wet chemistry methods, which allowed the control of their size, shape, and composition. Pd–Cu NCs were prepared according to modified one-pot procedures reported by Shan et al.^7^ and Yin et al.^28^ while Pt–Cu NCs were synthesized using the procedure reported by Yu et al.^29^ with minor modifications. In a typical synthesis of alloy NCs with an atomic Pd/Cu ratio equal to 50/50, 0.25 mmol palladium(II) acetylacetonate and 0.25 mmol copper(II) acetylacetonate were dissolved in 20 mL of benzyl ether. One mmol of 1,2-hexadecanediol dissolved in 5 mL of benzyl ether was added as the reducing agent. The mixture was heated slowly to 105 °C under N_2_ atmosphere, followed by the addition of 0.714 mL of OlAc and 0.741 mL of OlAm as capping agents to the as-formed dark solution. After the injection, N_2_ purging was stopped, and the temperature was increased to 220 °C keeping the reaction mixture at reflux for 30 min. The final product was cooled down to room temperature and transferred to the glovebox. The NCs were precipitated out by adding 25 mL of anhydrous isopropanol and centrifuging at 1000 rcf for 30 min. In the second washing step, 200 μL of anhydrous chloroform was used to wash the wall of the vials, then 100 μL of OlAm and 25 mL of isopropanol were added. After a second centrifugation at 1000 rcf for 10 min, the NCs were dispersed in anhydrous chloroform.

Pt–Cu NCs with an atomic Pt/Cu ratio of 50/50 were synthesized at room temperature by mixing 0.5 mmol of copper(II) acetylacetonate, 0.5 mmol of platinum(II) acetylacetonate and 5 mL of OlAm. The solution was heated to 280 °C at a rate of 5 °C min^–1^, and then it was cooled down to room temperature. The solution was diluted with 5 mL of ODE at 80 °C to avoid the agglomeration and the coalescence of NCs that may occur during the cooling. The black product was precipitated by adding 40 mL of ethanol and separated by centrifugation at 8421 rcf for 5 min. In the second washing step, 200 μL of toluene was used to wash the wall of the vials, then 40 mL of ethanol was added, and the NCs were precipitated by centrifugation at 8421 rcf for 5 min. Finally, the NCs were dispersed in toluene.

#### Monometallic Nanocrystals

Monometallic Cu, Pd, and Pt NCs were prepared and supported on γ-Al_2_O_3_ as a basis of comparison with the bimetallic ones. Cu NCs were synthesized using a method reported in the literature:^30^ 4 mmol of copper(I) acetate was mixed with 6.6 mmol of oleic acid and 15 mL of trioctylamine and degassed at 180 °C under an inert atmosphere of N_2_ for 1 h. Then, the solution was quickly heated to 270 °C and kept at this temperature for 15 min. The NCs were precipitated by adding 25 mL of ethanol and separated by centrifugation at 6654 rcf for 5 min, and then dispersed in hexane, generating a final green oxidized Cu_2_O nanocrystal solution. Pd NCs were prepared according to the procedure reported by Jin et al.^31^ Typically, a solution containing 0.33 mmol of palladium(II) acetylacetonate, 8 mL of ODE, and 10 mL of OlAm was heated to 100 °C under N_2_ flux. 0.33 mmol of MB dissolved in 2 mL of OlAm was added into the above solution. The resulting solution was heated to 130 °C and was further kept at this temperature for 20 min. Then, the solution was cooled to room temperature. The NCs were washed by adding 30 mL of anhydrous ethanol and precipitated by centrifugation at 1000 rcf for 5 min. The final product was collected and dispersed in hexane. Pt NCs were synthesized using the procedure reported in ref (32). In a typical synthesis, 0.13 mmol of platinum(II) acetylacetonate was mixed with 10 mL of OlAm. The mixture was heated to 100 °C under argon atmosphere. After 20 min, 3 mmol of TBAB dissolved in 5 mL of OlAm was injected into the above solution. The temperature was raised to 120 °C and kept for 1 h before cooling down to room temperature. For the washing procedure, repeated twice, 30 mL of ethanol were added, and the NCs were precipitated by centrifugation at 6654 rcf for 5 min. The final product was collected and dispersed in hexane.

#### Alumina-Supported Nanocrystals

Typically, a dispersion of γ-Al_2_O_3_ powder, in hexane for Cu, Pd, and Pt NCs, in chloroform for Pd–Cu alloy NCs and toluene for Pt–Cu alloy NCs, was sonicated for 5 min. A solution containing an appropriate volume of NCs was added to the support dispersion and left under fast stirring for 2 h. The powder was recovered by centrifugation at 1000 rcf for 5 min. The sample was finally dried in a vacuum oven at 40 °C for 1 h. The resulting powder was calcined at 450 °C for 3 h in a muffle furnace under static air to completely remove any capping ligand from the surface of the NCs. The calcination conditions were chosen based on the results of the thermogravimetric analysis (TGA) performed on colloidal NC solutions (Figure S1 of the Supporting Information, SI). The differences in the weight loss observed among the samples could be attributed to the variation in residual solvents and ligands uptake on the different samples.

#### Redox Treatments

The experiments were performed in a flow reactor consisting of a vertical quartz tube (6 mm internal diameter) where the calcined alumina-supported NCs were placed between two beds of quartz wool. All gases were introduced into the reactor via calibrated mass flow controllers, and a tubular furnace was used to heat the reactor. One thermocouple was placed inside the sample to monitor the temperature. The supported NCs were exposed to an oxidizing (6% v/v O_2_ diluted with He) and a reducing (5% v/v H_2_ diluted with He) atmospheres up to 350 °C for 1 h. The initial oxidation after loading was done to ensure a clean surface of the samples free of any adsorbed water or formed carbonates due to storage in an open environment. The heating rate used in both treatments was 5 °C min^–1^, and the total flow rate was 60 mL min^–1^.

### Nanocrystal Characterization Techniques

#### Transmission Electron Microscopy

The bright-field transmission electron microscopy (BF-TEM) images of NCs were recorded using a JEOL JEM-1011 instrument with a thermionic W source, operated at 100 kV. The samples were prepared by drop-casting the diluted NC solution or the alumina-supported NC powders suspended in hexane, toluene, or chloroform onto a carbon-coated 200 mesh Cu grids. High-resolution TEM images (HRTEM) were collected by a JEOL JEM-2200FS microscope (Schottky emitter), operated at 200 kV, equipped with a CEOS spherical aberration corrector for the objective lens, and an in-column Omega energy filter. The chemical composition of the NCs was determined by energy-dispersive X-ray (EDX) spectroscopy performed in the high-angle annular scanning TEM (HAADF-STEM) mode with a Bruker XFlash 5060 detector. The STEM-EDX maps were acquired using Cu Kα and Pd/Pt Lα lines, taking care to select signals only from the element of interest, without contributions from neighboring X-ray peaks. For HRTEM and STEM-EDX analyses, the samples were prepared by drop-casting NCs solutions onto ultrathin carbon-coated Ni grids. HAADF-STEM images of Cu NCs supported on γ-Al_2_O_3_ were acquired using a FEI Tecnai G2 F20 microscope (Schottky emitter), operated at 200 kV acceleration voltage. The selected area electron diffraction (SAED) patterns were acquired using the same microscope with a constant camera length, bringing the sample to eucentric height and eucentric focus. The camera length was calibrated using a nanocrystalline Au sputtered film on a standard carbon-coated Cu grid. The NC average size and distribution were obtained using ImageJ software.^33^ Alumina supported NCs were processed by manual counting using Gatan Digital Micrograph software from analysis of the HAADF-STEM images to determine the NC size distribution.

#### Inductively Coupled Plasma Optical Emission Spectroscopy (ICP-OES) Analysis

The measurements were carried on an iCAP 6000 Thermo Scientific spectrometer for quantification of the elemental composition of NCs and the metal loading. The samples (specific volume of NCs colloidal solution or weight of the alumina supported NC powder) were digested in aqua regia HCl:HNO_3_3:1 v/v (Sigma-Aldrich for trace analysis) overnight. Ultrapure Milli-Q water (18.2 MΩ cm) was added to the sample, and any remaining solids were filtered using a PTFE filter before the measurements. All chemical analyses were affected by a systematic error of about 5%.

#### X-ray Powder Diffraction (XRD)

XRD patterns of the NCs were collected on a PANalytical Empyrean X-ray diffractometer equipped with a 1.8 kW Cu Kα ceramic X-ray tube, PIXcel^3D^ 2 × 2 area detector and operating at 45 kV and 40 mA. The samples were prepared by dropping a concentrated NC solution or by directly depositing a powder onto a zero-diffraction silicon substrate. The diffraction patterns were performed at ambient conditions in a parallel-beam geometry and symmetric reflection mode over an angular range 30°–90°, with a step size of 0.05°. High Score 4.1 software from PANalytical was used for phase identification.

### In Situ Characterization Techniques

#### In Situ DRIFT

The measurements were performed using a Bruker Optics Vertex 70 FTIR spectrometer, equipped with a Praying Mantis DRIFT cell. Liquid nitrogen cooled Mercury Cadmium Telluride (MCT) detector was used for data acquisition and OPUS software for data processing. The outlet gaseous species were analyzed with a mass spectrometer (Pfeiffer Omnistar). A four-port selector valve was used to switch between two different gas streams, one used for the treatments and the other containing the CO probe. In a typical experiment, the DRIFT cell was loaded with 30 mg of the sample packed on top of about 80 mg of γ-Al_2_O_3_ (fine powder with particle size <63 μm). The supported NCs were treated under oxidizing and reducing atmospheres prior to the test, under the same gas compositions and temperatures described in the Redox treatments Section, except for the heating rate, which was set to 10 °C min^–1^, and the flow rate (80 mL min^–1^). The effect of changing the set parameters was verified and did not lead to different results (not reported here). The measurement sequence was the following: the sample was cooled to room temperature under a He flow, and a background spectrum was recorded at 25 °C directly after the treatment. After collection of the background, the He gas stream was switched to a stream containing 0.2% v/v CO balanced with He. Nine absorption spectra were collected every 10 s from the gas switch. After 6 min from the beginning of the adsorption process, additional 5 spectra were collected every 60 s, approaching the surface saturation. Then, the sample was purged with He and desorption spectra were acquired with the same frequency as in the adsorption phase.

#### In Situ XAS

Data were recorded in transmission mode at the Cu K-edge (8979 eV), Pt L_III_-edge (11564 eV) and Pd K-edge (24350 eV) on the ROCK (Rocking Optics for Chemical Kinetics) beamline of the synchrotron SOLEIL (France). For the measurements during the reduction/oxidation treatments, the calcined bimetallic supported NCs were exposed to 60 mL min^–1^ of a mixture of 5% v/v H_2_/He and then to 6% v/v O_2_/He. Spectral acquisition was also done on the samples in He at room temperature before each treatment for data comparison. Thanks to the edge jumping capability of the quick-scanning extended X-ray absorption fine structure (EXAFS) monochromator,^34^ both the edges of elements composing the NCs were characterized simultaneously. Indeed, Si(111) and Si(311) crystals were alternatively used as a monochromator for the Cu and Pd/Pt edges. Data were recorded for 1 min at one edge before changing the monochromator to the other one (with about 30 s of dead time for the exchange) and then keeping the subsequent minute at the other edge of interest. The cell allowed to perform in situ treatments of the supported NCs under controlled conditions such as temperature, pressure, and chemical environments during the experiment according to the assembled system described by La Fontaine et al.^35^ The calibration of the energy scale was ensured by the simultaneous measurement of the absorption spectrum of the correspondent metallic foil of the elements composing the NCs set between the second and third ionization chambers. The reference PtO, CuO, and Cu_2_O XAS spectra were measured in transmission mode at the SuperXAS beamline of the Swiss Light Source (SLS) at Paul Scherrer Institute (PSI Synchrotron). After normalization, the collected spectra were used as qualitative comparison of XANES part features with those of the pure XANES spectra of the formed compounds during the two treatments.

#### XAS Data Processing

The collected XAS spectra were initially extracted, subsequently calibrated and normalized by means of a graphical user interface (GUI) within Python developed at the ROCK beamline, as described in the literature.^36^ The normalized XAS data sets were then processed using the methodology of multivariate curve resolution-alternating least-squares (MCR-ALS) to unravel the number and the concentration profiles of the species evolving throughout the treatments. The number of variability sources, related to the number of chemical species, was analyzed using singular value decomposition (SVD), and evolving factor analysis (EFA).^37,38^ Assuming that the experimental data follow a linear model, this statistical analysis of minimization was carried out using the MCR-ALS GUI 2.0 developed by Tauler et al.^39^ on the Matlab platform, which decomposes the series of time-resolved spectra (D), recorded during reaction into pure species whose relative concentrations vary with time, as follows:
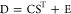
1where C is the matrix containing pure concentration profiles and S^T^ is the transposed matrix of S containing pure XAS spectra of the k species of the mixtures. E is the matrix of residuals, which contains the variability not explained by the model, ideally close to the experimental error. Non-negativity and unimodality constraints were applied on the matrices C and S, responsible for the observed data variance, to help the convergence of the multivariate curve resolution.

EXAFS extraction, Fourier transform (FT), and EXAFS simulation were performed using the software ATHENA and ARTEMIS within the Demeter package to obtain structural parameters: degeneracy of selected path (N) for each shell, interatomic distance (R), and Debye–Waller factor (σ^2^). Specifically, after data conversion into k space, the k^2^-weighted EXAFS functions were Fourier transformed and fitted in R space simulating the experimental signal. The fitting was performed for the first and second shell scattering at both edges to determine the identity, number and positions of the nearest neighbors and thus to generate the cluster around the selected absorbers. Initially, the ATOMS and FEFF packages implemented inside the program were employed to generate ab initio the scattering paths for the defined clusters starting from model compounds of known structure. The theoretical models for the Cu-substitute Pd and Pt alloys were built on structures containing 50% noble metal and 50% Cu atoms randomly distributed in a fcc lattice. In the case of Pd and Cu oxides phases, four model clusters were constructed around the Pd and Cu absorber atoms with respect to the structural data for PdO (ICSD 24692), Pd_2_O (ICSD 77651), CuO (ICSD 61323), and Cu_2_O (ICSD 26963), respectively. Finally, regarding the modeling of the clusters of the Cu_*x*_Pd_1–*x*_O phase, the PdO and CuO lattices in which the Pd and Cu absorber atoms were randomly replaced with the Cu and Pd atoms, respectively, were used as the initial structural data for the model. Then, a certain number of scattering paths were included in the modeling, fitting the k^2^-weighted EXAFS functions in the selected k range. The values of amplitude reduction factor (S_o_^2^) were obtained from fitting the standards (Cu, Pd and Pt foils) and their values were fixed in the analysis of the samples. The other parameters or variables, i.e., E_o_, N, R, and σ^2^ were obtained from the fittings. The reported Fourier transform of the EXAFS spectra and the best fits are not phase corrected.

## Results and Discussion

### NC Size, Composition, and Structure

**Figure 1:**
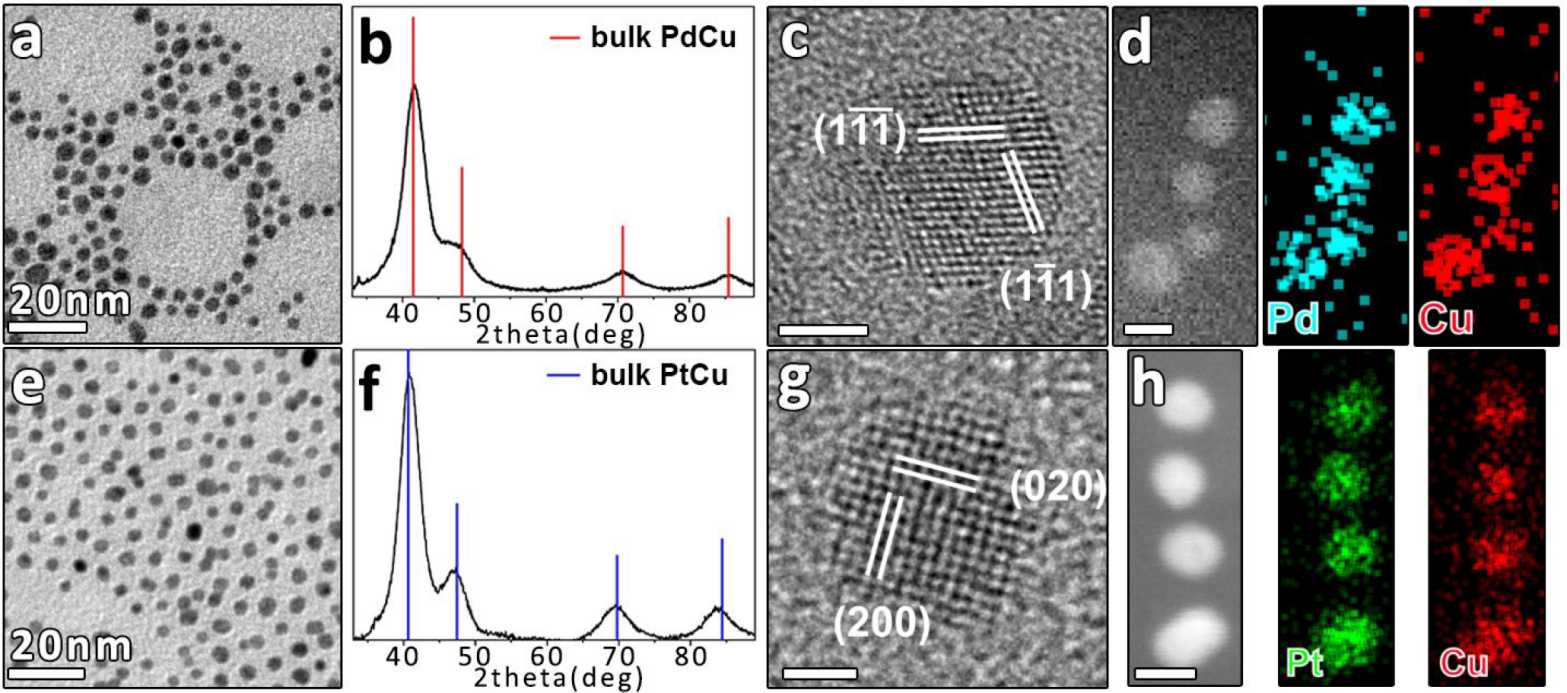
BF-TEM images of (a) Pd–Cu and (e) Pt–Cu NCs. XRD patterns of (b) Pd–Cu and (f) Pt–Cu NCs together with bulk reflections of cubic Pd–Cu (red bars, ICSD number 103082) and cubic Pt–Cu (blue bars, ICSD number 108402). HRTEM images of single-crystal (c) Pd–Cu and (g) Pt–Cu NCs, with the indication of lattice planes based on the same phases used for XRD analyses (see also Figure S3). The scale bars in (c) and (g) are 2 and 1 nm, respectively. HAADF-STEM images of (d) Pd–Cu and (h) Pt–Cu NCs with the corresponding EDX elemental maps. The scale bars in (d) and (h) are both 5 nm.

Typical BF-TEM images of spherical NCs with the corresponding size distribution histograms are reported in Figures 1a,e and S2. Table 1 summarizes the average size of NCs and, for the bimetallic alloy ones, their Cu content as measured by ICP-OES and STEM-EDX. All the NCs (except monometallic Pt and Cu with a size of 7–8 nm) had an average size of about 5 nm and the atomic fractions of Cu in the bimetallic NCs were matching the required target of noble metal:Cu = 50:50, within the experimental error. The element distribution within these alloy NCs was examined using HAADF-STEM imaging combined with EDX. Homogeneous distribution of Pd and Cu (Figure 1d) and of Pt and Cu (Figure 1h) within the NCs was clearly observed. HRTEM images of the alloy NCs evidenced mainly multiply twinned structures and, in some cases, single-crystal nanoparticles (Figure 1c,g). The corresponding fast Fourier transform (FFT) patterns (Figure S3a,b) were consistent with fcc structures with 3.77 and 3.78 Å lattice parameters for Pd–Cu and Pt–Cu NCs, respectively. These values are between the lattice parameters of Pd (3.89 Å) and Cu (3.62 Å), and Pt (3.94 Å) and Cu (3.62 Å), respectively. The XRD analysis (Figure 1b,f) confirmed the formation of phase-pure Pd–Cu and Pt–Cu solid solutions. Indeed, the obtained diffraction peaks in the XRD patterns shifted to higher angles compared to the pure phases of Pd and Pt (Figure S4e–f), respectively, due to the incorporation of Cu in the lattice. The lattice parameter, *a*, calculated using the interplanar distance *d*_111_ from the (111) diffraction peaks, was 3.75 Å for Pd–Cu and 3.80 Å for Pt–Cu, in agreement with what was observed by HRTEM analysis. Further evaluation of the Cu content in the alloy was made from the peak shift in the XRD pattern by assuming a linear relationship between the lattice constant of the alloy and the concentration of the constituent alloy (Vegard’s law).^40,41^ In the case of Pd–Cu NCs, the content of Cu in the alloy structure matched the amount of Cu in the sample, indicating fully alloyed NCs. Only a 29 atomic percentage (at.%) of Cu was found instead in the Pt–Cu NCs. Compared with the 50 at. % measured by ICP-OES and STEM-EDX (Table 1), this number could indicate the presence of an amorphous Cu phase not detectable by XRD. Thus, Vegard’s law cannot be applied correctly to this system as it is strictly valid for homogeneous alloys and unstrained particles.^42,43^

**Table 1:** Size and Chemical Composition for the As-Synthesized NCs^a^

sample	average size of NC as-synthesized (n° of measured NCs)	Cu at.% by ICP	Cu at.% by STEM-EDX	wt % metal load (NM + Cu)	average size after oxidation (n° of measured NCs)	average size after reduction (n° of measured NCs)
Pd_50_-Cu_50_	5.1 ± 1.5 nm (755)	53 ± 0.1^b^	53.3 ± 1.5^b^	1.9 ± 0.2^b^	7.8 ± 2.0 nm (86)	5.9 ± 1.6 nm (133)
Pt_50_-Cu_50_	4.9 ± 1.8 nm (1445)	51 ± 0.2^b^	46.8 ± 0.8^b^	2.0 ± 0.1^b^	4.7 ± 0.7 nm (201)	4.9 ± 0.6 nm (151)
Pd	5.2 ± 1.0 nm (1375)			1.9 ± 0.1^b^	5.5 ± 0.8 nm (85)	5.3 ± 0.8 nm (101)
Pt	7.6 ± 1.7 nm (174)			2.5 ± 0.2^b^	11.2 ± 4.8 nm (138)	8.0 ± 2.2 nm (88)
Cu	6.8 ± 1.7 nm (405)			0.8 ± 0.0^b^	26.4 ± 5.9 nm (72)	18.7 ± 4.4 nm (140)

a Metal load obtained from ICP-OES and the size after the two treatments for the mono- and bimetallic alumina supported NCs (NM = noble metal).

b Standard deviation of four repeated tests.

### Structure of the Supported NC after Redox Treatments

**Figure 2:**
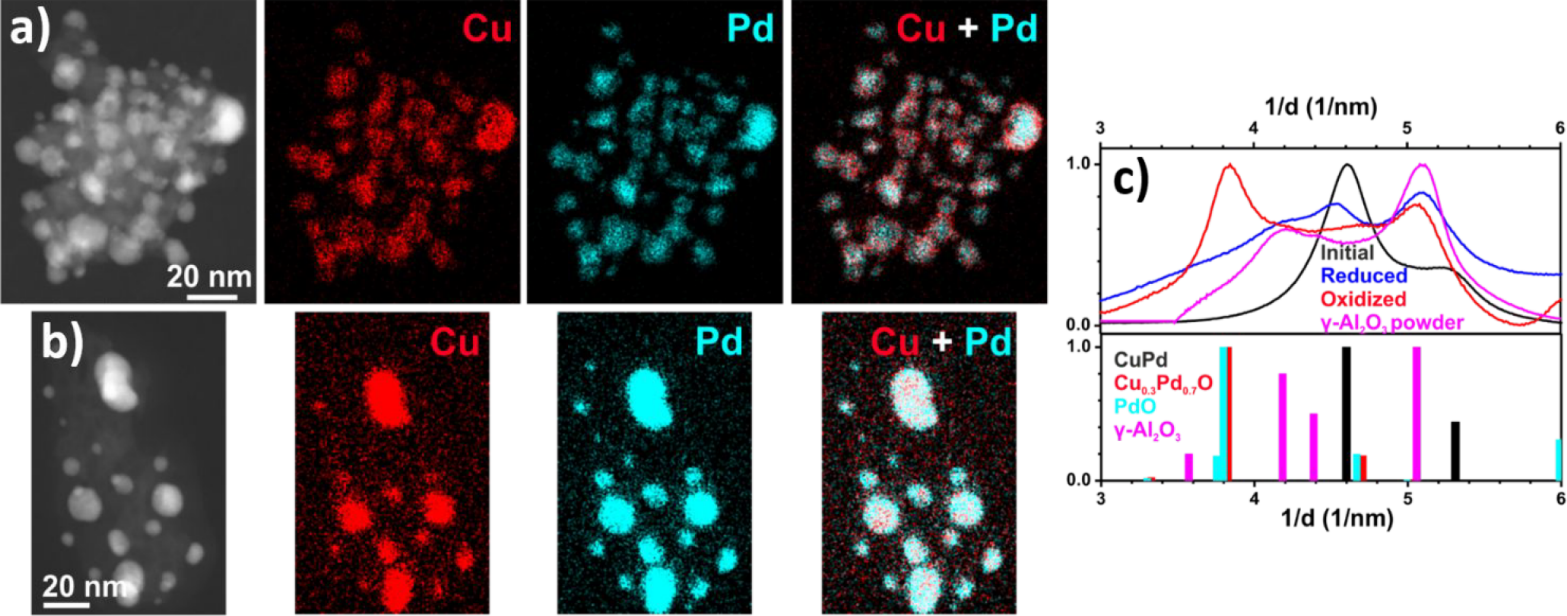
HAADF-STEM images of Pd–Cu alloy NCs supported on Al_2_O_3_ and corresponding EDX maps for Cu and Pd after (a) oxidizing and (b) reducing treatments; (c) azimuthally integrated, background-subtracted SAED patterns after oxidation and reduction in comparison to Pd–Cu NCs (reference patterns: Pd_1_Cu_1_ ICSD 103082 (black line), Pd_1_O_1_ ICSD 24692 (green line), and γ-Al_2_O_3_ ICSD 100425 (red line)). The reference pattern for Cu_0.3_Pd_0.7_O was calculated by modifying the tetragonal cell of PdO (ICSD 24692). The new cell had Cu and Pd ions with fractional occupancies (0.3 and 0.7, respectively) in the sites of Pd ions and parameters, *a* = *b* = 3.005 Å and *c* = 5.29 Å, calculated according to the plots in ref (45) for Cu_*x*_Pd_1–*x*_O, with *x* = 0.3).

**Figure 3:**
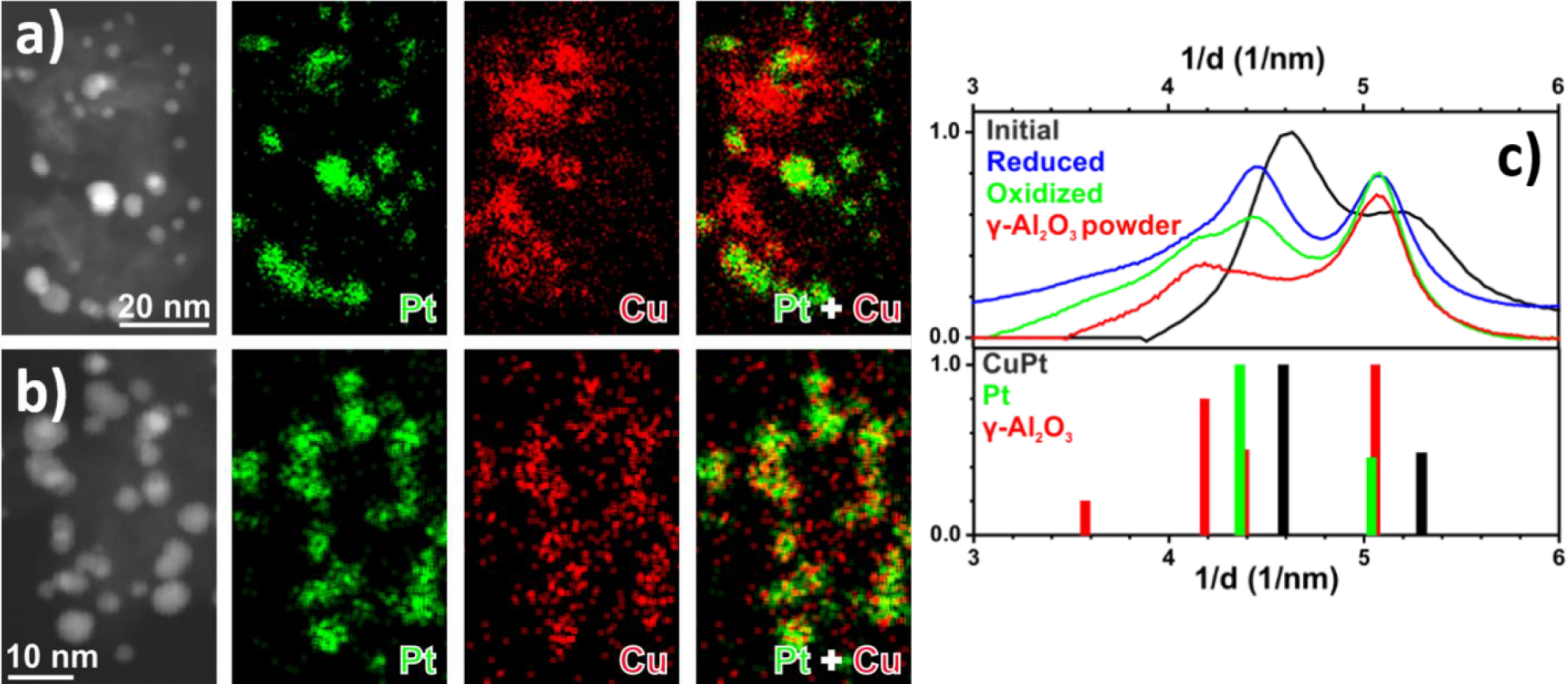
HAADF-STEM images of Pt–Cu alloy NCs supported on Al_2_O_3_ and corresponding EDX maps for Pt and Cu after (a) oxidizing and (b) reducing treatments; (c) azimuthally integrated, background-subtracted SAED patterns after oxidation and reduction in comparison to Pt–Cu NCs (reference patterns: Pt_1_Cu_1_ ICSD 108402 (black line), Pt ICSD 41525 (green line), and **γ**-Al_2_O_3_ ICSD 100425 (red line)).

The total metal loads measured by ICP-OES of NCs supported on γ-Al_2_O_3_ and subjected to oxidative/reductive treatments at 350 °C are reported in Table 1. To track the NC structural changes under different gas atmosphere conditions, the NCs were characterized by TEM after treatments. The HAADF-STEM images of supported Pd–Cu and Pt–Cu NCs are shown in Figure S5. The Pt–Cu NCs were homogeneously distributed on the support, regardless of the treatments, with no NC morphology change; their average size remained unchanged (Table 1) and about equal to that of the as-synthesized NCs. Pd–Cu NCs were also well dispersed on the grains of the alumina support after both oxidizing and reducing treatments, confirming that no significant sintering phenomena had occurred with time on stream. However, it is not possible to completely exclude the formation of a small fraction of large particles possibly related to the sintering that occurred during calcination. However, a significant increase of the average size of the Pd–Cu NCs to about 53% was observed after oxidation, mainly due to the formation of a flocky shell on the NC surface, poorer in Pd than the NC core, as evidenced by the STEM-EDX analysis (Table 1 and Figures 2, S5a, and S6). Relative to the size of the supported monometallic NCs, no significant changes were observed in the case of Pd NCs after the two treatments, as well as for Pt NCs after reduction. After oxidation, the Pt NC size increased by 47%. A notable change was especially found for oxidized and reduced Cu NCs with triple and double size increase, respectively (Figure S7a,b and Table 1). This can be explained as a result of NC agglomeration induced by reactants and/or sintering phenomena under redox treatments by coalescence of smaller NCs or by Ostwald ripening,^44^ which were altered in the presence of a second metal. Nevertheless, the presence of larger NCs in the as-synthesized Cu NCs could enhance the above-mentioned process, due to the detachment of metal atoms from small particles with high chemical potential as transient monomers, diffusion onto the support as oxygen–metal complexes, and subsequent attachment to larger particles with lower chemical potential. The bimetallic NCs were analyzed by STEM-EDX to study the spatial distribution of the elements after the two treatments. After oxidation, STEM-EDX maps showed that Cu was predominantly associated with Pd within the NCs, and only a small amount of Cu had diffused onto the alumina support. In particular, larger NCs exhibited a Cu-rich shell surrounding a Pd-rich core (Figure 2a). The comparison between EDX spectra (not shown) extracted from two identical areas in the map in Figure 2a, one from the shell and the other one from the core region of one of NCs on the surface of the alumina support fragment, returned a Cu/Pd atomic ratio of 0.8 in the NC shell region, to be compared to 0.3 in the NC core. However, while Pt was still localized in NCs entities, a higher concentration of Cu was detected from all over the alumina support (Figure 3a), as found for Au–Cu NCs on Al_2_O_3_.^25^ After reduction, Pd and Cu were localized in the same region of the Pd–Cu NCs, as well as Pt and Cu in the Pt–Cu NCs, with only a weak Cu signal detected on the support (Figures 2b and 3b), as in the case of the Au–Cu NCs.^25^ Due to the low metal loading, the small NCs, and the presence of γ-Al_2_O_3_, the XRD profiles obtained after the treatments were mostly dominated by the support contribution, thus limiting their interpretation. For this reason, SAED patterns of supported Pd–Cu and Pt–Cu NCs were collected after the treatments and compared with the pattern of the as-synthesized NCs (Figures 2c and 3c). Since the peaks of γ-Al_2_O_3_ partially overlapped with the peaks of the NCs, the SAED pattern of pure γ-Al_2_O_3_ is also reported. After oxidation of the supported Pd–Cu NCs (Figure 2c), the collected SAED pattern was consistent with the formation of a Cu_0.3_Pd_0.7_O solid solution. This assertion was justified by the fact that the diffraction peaks detected were slightly shifted to higher 2θ values compared to the PdO reference pattern due to the random replacement of Pd^2+^ with Cu^2+^ in the Pd oxide lattice.^45^ This assignment was confirmed by STEM-EDX, in which an average atomic composition of Pd:Cu = 2.4:1, and by HRTEM analysis, which agrees with a Pd_0.7_Cu_0.3_O solid solution (Figure S8). On the basis of these results, it was possible to estimate whether the measured increase of NC size could be ascribed to the formation of Pd_0.7_Cu_0.3_O NCs. From the experimental data, the average size of the starting Pd_0.5_Cu_0.5_ NCs was 4.9 ± 1.8 nm, while that calculated after oxidation with eq S3 was 7.0 nm, a value in agreement with that observed in the HAADF-STEM images (7.8 ± 2.0 nm). Thus, an increase in NC average size of 43% was observed compared to the initial NC size. After reduction, the supported Pd–Cu NCs pattern (Figure 2c) showed characteristic peaks related to the fcc phase of Pd–Cu. Indeed, the comparison with the pattern of the as-synthesized NCs suggested that the Pd–Cu alloy composition with 36 at. % of Cu (estimated by Vegard’s law)^40^ was restored. The SAED pattern of Pt–Cu/Al_2_O_3_ after oxidation (Figure 3c) featured diffraction peaks that were characteristic of a cubic structure, with a 3.9 Å lattice parameter corresponding to Pt; no Cu or CuO_*x*_ peaks were detected. By coupling this information with the STEM-EDX maps, we could conclude that Cu was highly dispersed onto the support either as an amorphous phase or as a finely dispersed phase. The reduced Pt–Cu/Al_2_O_3_ SAED pattern (Figure 3c) resembled the one after oxidation, with no significant shift of the peak positions toward higher 2θ angles due to the reincorporation of Cu into the NCs.

Indeed, the obtained lattice spacing of 3.9 Å was comparable to that obtained after oxidation, suggesting no difference between oxidized and reduced Pt–Cu NC samples. This result can be explained by considering that it is not possible to rule out the exposure of the reduced sample to air when unloaded from the reactor. For this reason, as soon as the system was exposed to air, it was immediately oxidized. Additional experimental proofs for this conclusion will be provided in the next sections describing the EXAFS results. Therefore, the calculated value of Cu incorporation in the alloy after reduction (12 at. % from Vegard’s law) represented a rough estimate of the state of the Pt–Cu system after this treatment.

### NC Surface Chemistry Evolution

**Figure 4:**
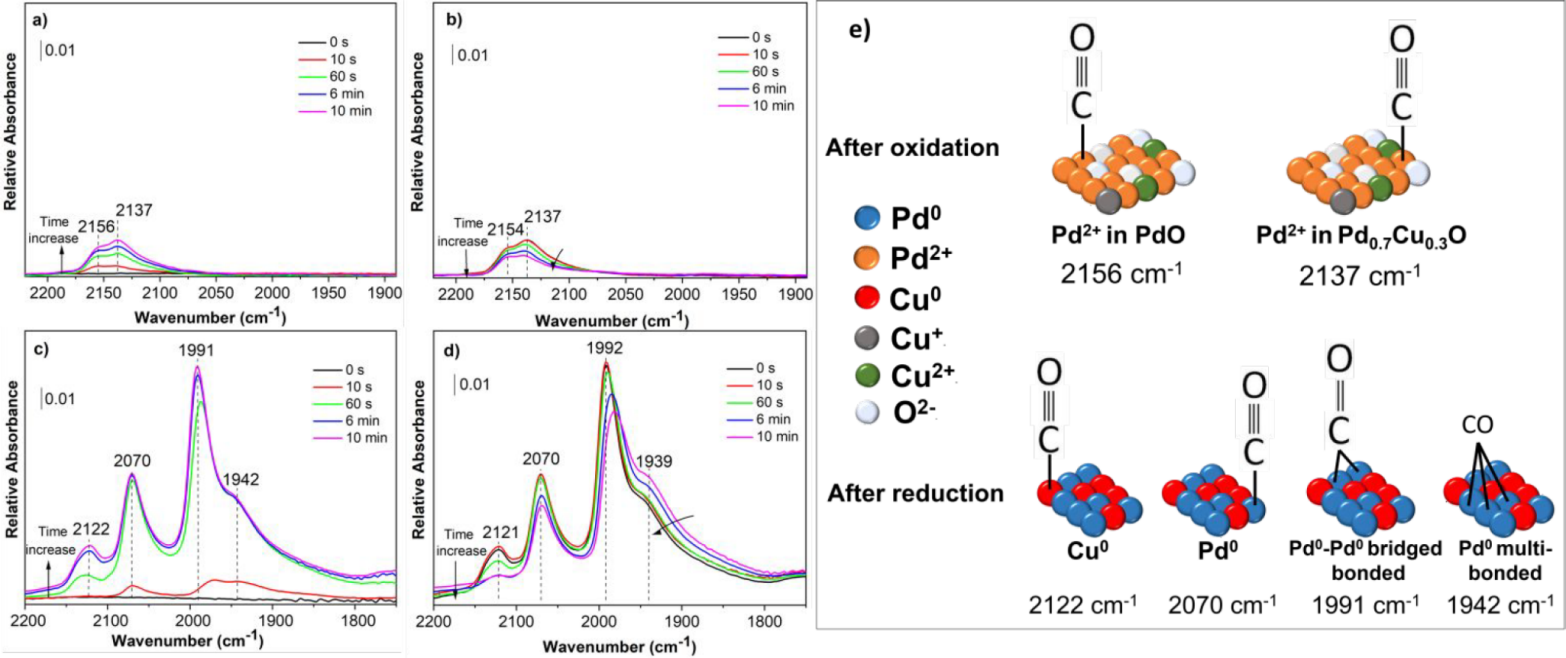
DRIFT spectra in the carbonyl region recorded during the adsorption (a, c) and desorption (b, d) of CO at room temperature on Pd–Cu alloy NCs supported on Al_2_O_3_ after the (a, b) oxidizing and (c, d) reduction treatments; (e) schematic illustration of infrared band assignments for the carbonyl species on Pd–Cu NC identified in the DRIFT spectra.

**Figure 5:**
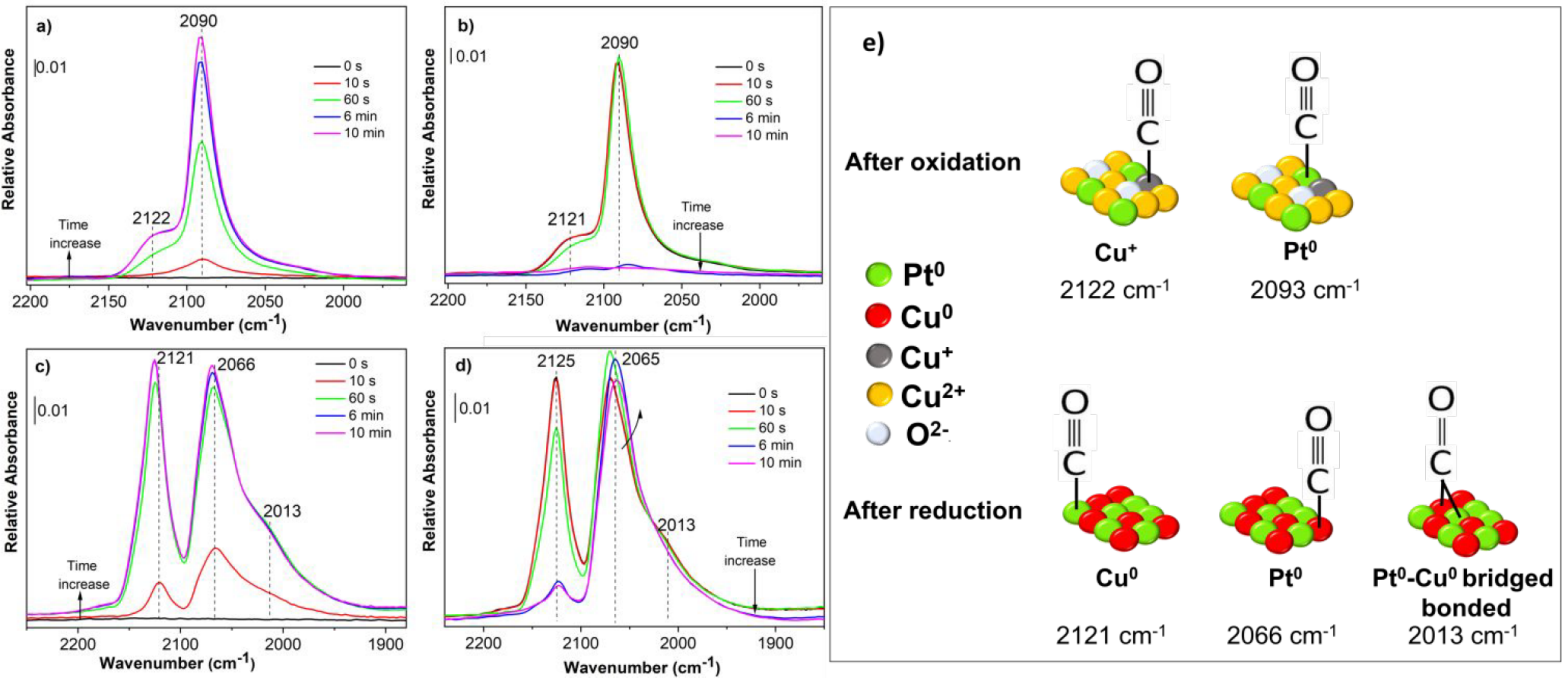
DRIFT spectra in the carbonyl region recorded during the adsorption (a, c) and desorption (b, d) of CO at room temperature on Pt–Cu alloy NCs supported on Al_2_O_3_ after the (a, b) oxidizing and (c, d) reduction treatments; (e) schematic illustration of infrared band assignments for the carbonyl species on Pt–Cu NC identified in the DRIFT spectra.

DRIFT tests on supported bimetallic NCs after oxidizing and reducing treatments were performed to probe the NC surface composition and obtain information about the electronic features of the metals in NC surface. It was found that the different sequences of reduction and oxidation on calcined supported bimetallic NCs do not influence the spectra in terms of number and position of the bands observed (not reported), meaning that the surface transformations are fully reversible. In addition, the effect of oxidative treatment with or without the initial calcination, which was typically done to remove the capping agents, was investigated and no difference was found (not shown here). This proved that the in situ oxidation treatment was also efficient in removing the capping agents and induced the same transformation when O_2_ is present in the atmosphere. Figures 4 and 5 report the DRIFT spectra in the carbonyl region at different exposure times during room temperature absorption and desorption of CO on/from the two bimetallic NCs. Under the same conditions, CO adsorption spectra for supported Cu, Pd, and Pt NCs were also acquired (Figures S9, S11, and S12). The CO adsorption on the oxidized Pd–Cu NCs (Figure 4a,b) resulted in the appearance of two overlapping bands at 2153 and 2137 cm^–1^ (note the difference in the intensity compared to the reduced ones). As it is reasonable to assume that the Cu is mostly oxidized to Cu^2+^ at the conditions of the oxidative treatment, and since carbonyl on Cu^2+^ are not stable and not detectable at room temperature,^46^ we assigned these two main bands to the carbonyl on Pd^2+^ species. The difference in the band positions could be attributed to the Pd adsorption sites in different coordinating environments, i.e., either localized as PdO phase or in close proximity with the Cu in the Pd_0.7_Cu_0.3_O phase. However, we could not rule out the presence of Cu^+^ species with respect to the data recorded on the monometallic Cu NCs (Figure S9a,b). To have a better band separation, the 10 min spectrum of oxidized PdCu was magnified and deconvoluted, which resulted in 5 bands (Figure S10a). The major components at 2153 and 2131 cm^–1^ could be assigned to carbonyl on Pd^2+^, in PdO and Pd_0.7_Cu_0.3_O phases, respectively, as stated above. The minor band 2102 cm^–1^ could then be attributed to carbonyls on Cu^+^ in close contact with Pd.^47,48^ The above description is consistent with a prior investigation of supported Pt–Cu NCs that highlighted how the atomic closeness of Cu atoms with Pt ones affected the mutual environment and, thus, the positions of the bands.^49^ The presence of Cu^+^–CO species could be inferred from the stability of this band during the desorption at room temperature (see the deconvoluted 10 min spectrum in Figure S10b), although, since the desorption at high vacuum and over a longer time could not be performed, bands on Pd^2+^ were still present.

The Cu^+^–CO band position in the CO desorption spectrum was shifted to 2110 cm^–1^ due to the strong dynamic coupling between the adsorbed molecules with increasing CO surface coverage.^46^ The additional and very low-intensity bands at 2069 and 1990 cm^–1^ could be related to linearly bonded and bridge-bonded Pd^0^–CO, respectively, suggesting partial oxidation of the supported Pd–Cu NCs, as found for the monometallic ones (Figure S11a), possibly due to a temperature deviation of the sample inside the cell vs the set point. In addition, the high-frequency band at 2153 cm^–1^ which we assigned to CO linearly adsorbed on Pd^2+^ atoms, could be corroborated by performing the same experiment on the monometallic Pd NCs (Figure S11a,b).^46^ After reduction (Figure 4c,d), three main bands centered at about 2122, 2070, and 1991 cm^–1^ with a shoulder at 1942 cm^–1^ appeared. The three latter vibrational bands can be straightforwardly attributed to CO bonded to surface Pd atoms in the linear, bridged and 3-fold hollow bonded forms, respectively.^50^ As the surface coverage increased by exposure time, the CO stretching frequency of Pd-bounded CO shifted to a higher frequency (about 10 cm^–1^) due to the dipole–dipole coupling between adsorbed CO molecules on Pd surface. Although the frequency of the band at 2122 cm^–1^ was in the range of CO adsorbed on Cu^+^ (2110–2140 cm^–1^), the attribution of this band to CO adsorbed on Cu^0^ can be justified by assuming an electronic modification by neighboring Pd atoms that increases the CO–Cu^0^ strength and, thus, shifts the band position. Indeed, the frequencies of the bands assignable to linearly bonded Pd^0^–CO and Cu^0^–CO carbonyls in the spectra of reduced Pd (Figure S11c,d) and Cu (Figure S9c,d) NCs were about 16 cm^–1^ higher and 4 cm^–1^ lower, respectively, than those recorded for the bimetallic NCs. This confirmed the presence of an electron transfer from Cu to Pd such that the electronic properties of Pd atoms were strongly modified by Cu addition and vice versa. After 10 min of evacuation (Figure 4d), the incomplete disappearance of the band at 2122 cm^–1^ implied that the Pd–Cu NCs might not be fully reduced due to the presence of residual Cu^+^ species on which CO was strongly adsorbed in addition to reduced Cu^0^. This was in agreement with SAED characterization, which suggested that the copper was not completely reincorporated into the alloy. However, as in the case of the oxidized sample, the incomplete disappearance of the bands could be related to the fact that the desorption could not be performed either under higher vacuum or for a longer time. The schematic visualization of the band assignment of carbonyl species on Pd–Cu system is reported in Figure 4e.

CO adsorbed on the oxidized Pt–Cu NCs (Figure 5a,b) resulted in a main absorption band at 2090 cm^–1^ and a second weak band at 2122 cm^–1^. The former was attributed to the linear CO species on Pt atoms^46^ and the latter to those on Cu^+^ atoms,^51^ in agreement with what obtained for monometallic Pt (Figure S12a,b) and Cu (Figure S9a,b) NCs. One should note that most of the Cu atoms are reasonably oxidized to the 2+ state, and the presence of Cu^+^ could be due to the deviation of the temperature in the DRIFT cell and the temperature set point. The spectra after reduction (Figure 5c,d) exhibited main CO bands at about 2121 and 2066 cm^–1^ with a shoulder at 2013 cm^–1^. CO adsorption on metallic Pt (Figure S12c,d) led to vibrational stretches in the range 2080–2098 cm^–1^ assigned to the CO linearly adsorbed on low-index planes (terraces) of the NCs, while the lower ones (2060–2075 cm^–1^) to the oscillation of CO molecules linearly adsorbed on Pt steps, edges, and corners.^52^ In this way, the band around 2066 cm^–1^ was attributed to this latter species. The high-energy band at 2121 cm^–1^ resulted from CO bound to Cu^0^, even if the position of this band would be more consistent with Cu^+^ rather than Cu^0^.^53^

The blue-shift of about 28 cm^–1^ of the frequency of CO on Pt in Pt–Cu NCs compared with that in monometallic Pt NCs (Figure S12c) was attributed to the electronic interaction between the two metals and a decreased dipole–dipole coupling due to the dilution of Pt by Cu.^54^ Likewise, the frequency of CO on Cu^0^ in the bimetallic NCs was about 4 cm^–1^ higher than the one recorded for reduced Cu NCs (Figure S9c). Indeed, this effect enhanced the electron density on Pt atoms ensuring that, during the evacuation, the adsorption of CO on Pt was much stronger than that on Cu.^55^ Furthermore, the evidence that the Pt and Cu were atomically mixed in the NCs was provided by two effects visible in the spectra: the first one was associated with the appearance of the band at 2013 cm^–1^ related to CO bridged between Pt and Cu atoms;^56^ the second was visible in the spectra during the evacuation (Figure 5d) in which the intensity of the Pt^0^–CO band increased at the expense of that of Cu^0^–CO due to an energy intensity redistribution effect.^49^ The infrared band assignments for the Pt–Cu system are summarized in Figure 5e.

### NC Geometric and Electronic Structural Modifications

XAS was applied to obtain information about the local geometric and/or electronic structure of NCs in response to the variation of the gas environments and temperature. In this regard, the transformations of Pd–Cu and Pt–Cu NCs were in situ monitored at the Pd K-edge, Pt L_III_-edge, and Cu K-edge X-ray absorption near edge structure (XANES) spectra while the NCs were subjected to the reducing and oxidizing treatments. The overview of all XANES spectra recorded during the experiments is presented in Figures S14 and S15. Initial XAS spectra were recorded at the Pd K-, Pt-L_III_-, and Cu K-edges of the as-synthesized Pd–Cu and Pt–Cu NCs (not shown). The results of the EXAFS analysis for Pd–Cu NCs (Tables S1 and S2) indicated the presence of alloy nanoclusters by the detection of the Pd–Cu_1_ and Cu–Pd_1_ coordination signals at 2.63 and 2.65 Å, respectively, for both Pd- and Cu K-edges, in agreement with what observed by STEM-EDX, HRTEM, and XRD analysis. For Pt–Cu NCs, the fitting of EXAFS (Tables S3 and S4) indicated the formation of Pt–Cu alloy nanoclusters at both edges along with the formation of the CuO phase at the Cu K-edge. A higher total coordination number (CN) for Pt (Pt–Pt_1_ and Pt–Cu_1_ 9.08) compared to that one for Cu (Cu–Cu_1_ and Cu–Pt_1_ 3.28) was found in the Pt–Cu alloy, indicating that Cu was preferentially segregated. This result can be justified considering a possible alloy inhomogeneity or a beginning of metal segregation due to the difference in atomic radius between Pt (1.39 Å) and Cu (1.28 Å).

XAS spectra of alumina-supported Pd–Cu and Pt–Cu NCs were collected in He as the starting point of the measurement (Figure S13). The Pd and Cu K-edge XANES spectra of Pd–Cu NCs were similar to those of PdO and CuO standards (Figure S13a,b). The EXAFS analysis (Tables S1 and S2) confirmed the presence of PdO and CuO along with the formation of PdCuO mixed oxides species with Cu–O and Pd–O as first neighbors. In the case of the Pt–Cu sample, the Pt L_III_ absorption edge laid in between those of Pt and PtO standard ones and the Cu K-edges spectrum was similar to that of the CuO standard (Figure S13c,d). Structural parameters obtained from the fitting of EXAFS at both edges (Tables S3 and S4) confirmed the formation of PtO, with a major contribution of metallic Pt and CuO. These results were consistent with the oxidation occurring during the calcination step, necessary for the removal of the organic ligands from the NCs surface, during which NC dealloying occurred.

**Figure 6:**
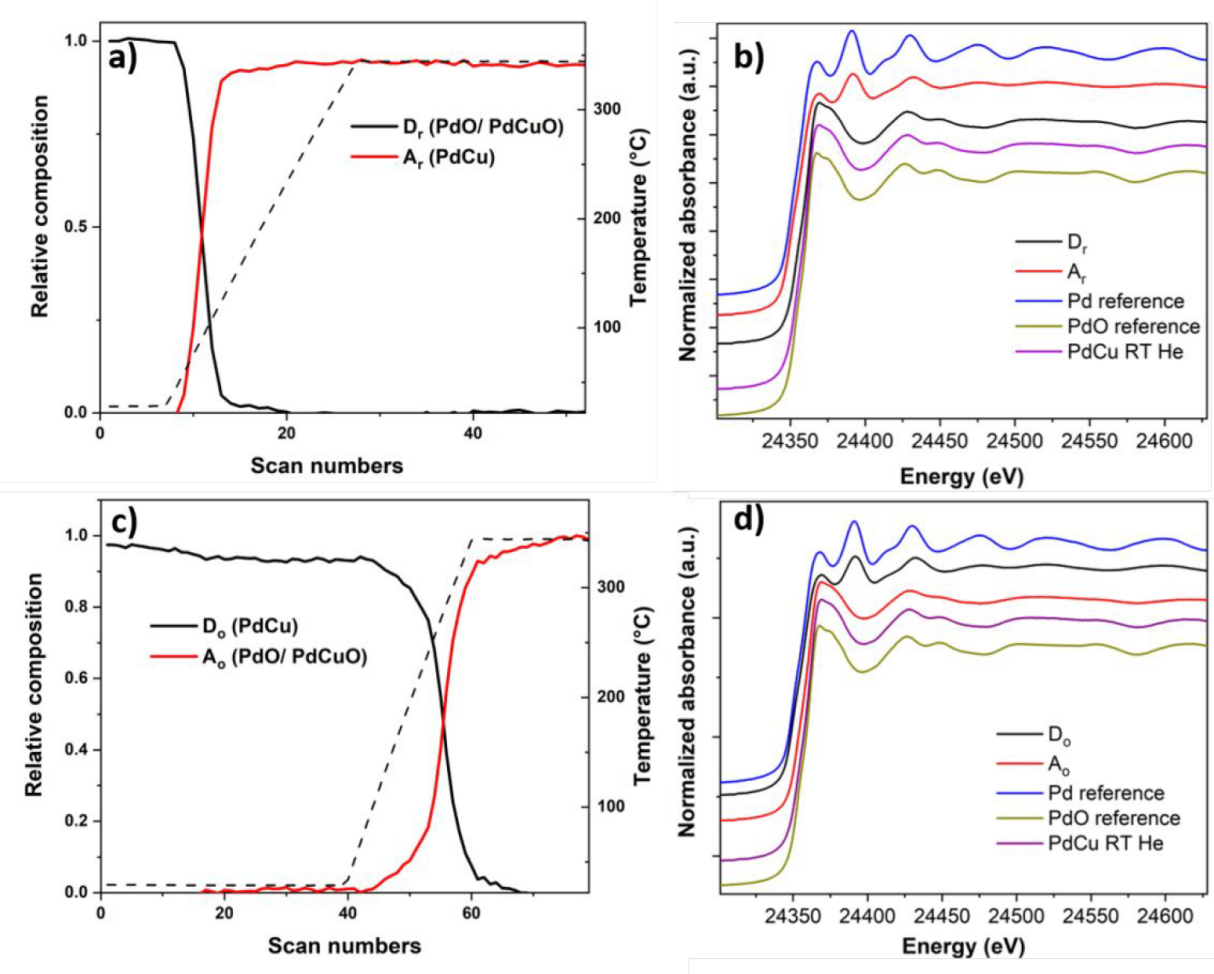
Concentration profiles of principal components as a function of the temperature during reduction (a) and oxidation (c) at the Pd K-edge. XANES spectra of the species formed during reduction (b) and oxidation (d) for the supported Pd–Cu NCs along with the XANES spectra of Pd and PdO references and supported Pd–Cu NC spectrum collected at room temperature in He.

The concentration profiles of the two obtained principal components named D_r_ (descending component, i.e., the concentration of which was decreasing) and A_r_ (ascending component, i.e., the concentration of which was increasing) formed during the reduction treatment at the Pd K-edge of the Pd–Cu NCs are reported in Figure 6a,b. The composition profile evidenced a significant reduction of the component D_r_ and a corresponding increase of the component A_r_ at *T* > 50 °C. By comparing the D_r_ and A_r_ component spectra (Figure 6b) with different standards, it is possible to claim that the D_r_ spectrum displayed XANES edge features resembling those of the PdO reference,^57^ while the A_r_ spectrum showed features similar to the metallic Pd reference. From the fitting procedure of the Fourier transform (FT) of the EXAFS spectra of the two components D_r_ and A_r_, the D_r_ is described by two major contributions corresponding to the Pd–O and Pd–Pd coordination shells at distances of 2.03 and 3.06 Å, related to PdO (Table S1). The additional formation of PdCuO mixed oxide-like was only observed during the EXAFS data refinement. The best EXAFS fitting for the A_r_ component was instead obtained with a Pd–Pd contribution at 2.70 Å and Pd–Cu one at 2.64 Å, characteristic of Pd–Cu. The large contribution of Pd–Pd distances found in the first coordination shell suggested the formation of a Pd-rich Pd–Cu disordered alloy. This observation is in line with the results obtained from the SAED data, in which the composition of the reduced NCs does not fully match with the starting NC alloy, while a Pd-rich alloy was instead found.

During the oxidative treatment at the Pd K-edge, the evolution of the concentration profiles of the two bimetallic NCs as a function of temperature, along with their pure spectra, are presented in Figure 6c,d. Specifically, the concentration of the component D_o_ started to decrease gradually at about 150 °C under exposure to oxygen and becoming increasingly evident during the heating ramp in the oxidation atmosphere with a simultaneous increase of the component A_o_. An initial visual inspection of the pure XANES spectra of the components with respect to the references ones (Figure 6d) showed that the shape of the features of the XANES part of D_o_ was consistent with what has been seen in bulk Pd–Cu^57^ while the A_o_ component features were similar to those of the PdO reference. The qualitative description of these two principal components was then corroborated by the analysis of the FT of Pd K-edge EXAFS oscillation of the representative components during oxidation. The best agreement between the observed and calculated EXAFS for D_o_ was achieved by using a structural model derived from the Pd–Cu alloy. In particular, a Pd-rich Pd–Cu disordered alloy was obtained from the fit. The component A_o_ was instead fitted using the structural models of PdO and PdCuO mixed oxide (Table S1).

**Figure 7:**
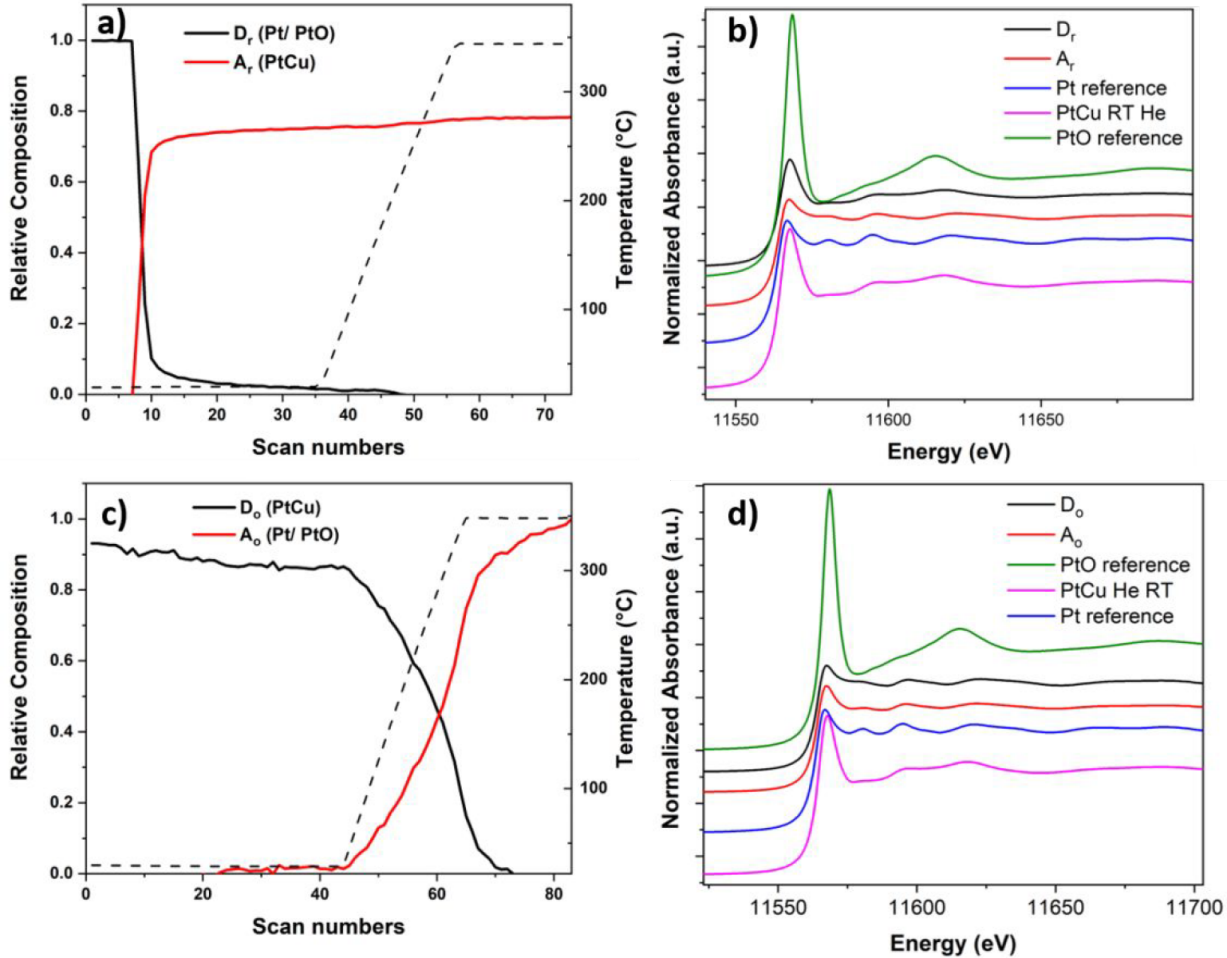
Concentration profiles of components as a function of the temperature during reduction (a) and oxidation (c) at the Pt L_III_-edge. XANES spectra of the species formed during reduction (b) and during oxidation (d) for the supported Pt–Cu NCs along with the XANES spectra of Pt foil reference, PtO reference spectrum collected at SuperXAS beamline of the SLS and supported Pt–Cu NC spectrum collected at room temperature in He.

In situ XAS at the Cu K-edge confirmed the Cu reduction and partial realloying under reducing conditions and then the dealloying during the oxidative treatment (see Figure S16 in the SI for additional details). To summarize, the fitting carried out at the Pd- and Cu K-edges during reduction indicates the formation of PdO and CuO with a minor contribution of PdCuO mixed oxide at the initial stage of the reduction. As the temperature was increased, Cu_2_O and Pd–Cu alloy were formed as intermediate species. The presence of Cu^+^ as an intermediate is commonly evidenced by XAS as was found for the systems in which Cu is supported on Al_2_O_3_, SiO_2_, and ZrO_2_ compared to the bulk powders in which the direct reduction of CuO to metallic Cu is reported.^58,59^ Finally, Pd-rich Pd–Cu disordered alloy and metallic Cu with a minor contribution of Cu_2_O were formed in the last step of the reduction, indicating that the initial alloy structure was not fully recovered. During the oxidation, the Pd-rich Pd–Cu alloy was already segregated at room temperature, with the formation of Cu_2_O as intermediate species to yield PdO, CuO, and, to a minor extent, a PdCuO mixed oxide. A heat treatment at 350 °C was required to fully oxidize the Cu oxide species in the +2 oxidation state, restoring the system to the initial situation recorded after the calcination. This outcome is partially in agreement with the picture obtained by DRIFT measurements. Indeed, due to the DRIFT setup limitations and the intrinsic deviation from the actual temperature set point reached inside the cell at high temperatures, complete oxidation of Cu was not observed under DRIFT. In addition, one should note that the DRIFT experiment is more surface-sensitive while the XAS elucidates the bulk properties, which could be overlooked by the DRIFT. XAS data were also examined at the Pt L_III_- and Cu K-edges for the alumina-supported Pt–Cu NCs during the oxidative and reductive treatments. On the basis of the concentration plot of the principal components during the reduction at the Pt L_III_-edge (Figure 7a), the component D_r_ rapidly decreased already at room temperature under the exposure to H_2_ with the consequent increase of the A_r_ component. By comparison with the references XANES spectra (Figure 7b), the D_r_ spectrum visually resembled the Pt one with a characteristic shape and edge shifted to higher energy with respect to the Pt reference one,^60^ to which the A_r_ spectrum approached. From the FT of the EXAFS part (Table S3), the best fit of the D_r_ EXAFS data confirmed the assignment to metallic Pt (CN 4.24 ± 0.6 at 2.8 Å) with a minor contribution of PtO (first shell CN 1.79 ± 0.0.2 at 2.0 Å) at the initial stage of the reduction, as already observed for the supported NCs after calcination. Referring to the A_r_ component, the high coordination number of Pt around Pt atoms in the first shell of Pt–Cu structure (CN 8.00 ± 0.6) suggested the formation of Pt-rich Pt–Cu alloy in the second stage of the reduction. The formation of these species during the exposure of the sample to a reductive environment was confirmed by the results obtained at the Cu K-edge (see Figure S17a,b in SI for more details). Two principal components were chosen to explain the variance of the data set during the oxidative treatment at the Pt L_III_-edge. From the concentration profile (Figure 7c), the descending D_o_ component was found to be stable and present at room temperature under exposure to oxygen.

At the beginning of the heating ramp, the D_o_ decrease was associated with the appearance of the ascending A_o_ component. The two components showed similar XANES features related to metallic Pt by comparison with the standard Pt (Figure 7d). In particular, the A_o_ edge was shifted slightly to lower energies, indicating stronger metallic features.^61^ Despite the similarity found by the visual inspection of the XANES spectra, the fit of the FT EXAFS part of the two components suggested an assignment to different species. Specifically, the presence of Pt-rich disordered Pt–Cu alloy at the initial stage of the oxidation as component D_o_ was evident from the presence of the Pt–Pt_1_ first shell at 2.72 Å with a coordination number of 6.96 ± 0.8 and Pt–Cu_1_ one at 2.68 Å with a CN of 2.83 ± 0.7. The fitting of the A_o_ FT EXAFS spectrum indicated that the Pt atom was surrounded by nine Pt atoms at a distance of 2.73 Å with a minor contribution of the PtO phase (CN Pt–O 0.36 in the first shell). The dealloying process was confirmed by the Cu K-edge data as well (see in Figure S17c,d the SI for additional details). To sum up, two principal components were found to be relevant for the supported Pt–Cu NCs during the exposure to H_2_. Metallic Pt and a minor fraction of PtO mixture phases were transformed into a Pt-rich Pt–Cu alloy already at room temperature, which remained unchanged until the end of the process. The formation of this latter species was in agreement with the results obtained from the SAED analysis, in which the initial NC alloy was found not to be fully restored. Complete segregation occurred during the oxidation treatment starting from a Pt-rich Pt–Cu alloy to metallic Pt and CuO phases at the end of the process, as confirmed by the previous techniques. Considering the previous results, it is possible to claim that, as for the case of the Au–Cu NCs, in which the CuO_*x*_ was found as isolated species away from the Au NCs on the support after the oxidizing treatment,^25,26^ the same situation was observed for the Pt–Cu NCs. This is different from the Pd–Cu NCs case, in which the oxidized Cu was retained by the palladium with the formation of PdCuO mixed oxide, along with a small fraction of CuO_*x*_. In general, during the reduction, the CuO_*x*_ species, found around the NCs or dispersed on the support, were partially realloyed within the Pt NCs to a minor extent compared to the Pd–Cu NCs. Indeed, 36 at. % of Cu was found in the Pd–Cu NCs on Al_2_O_3_ versus 12 at. % for Pt–Cu on the same support. Note that the latter value reported for Pt–Cu could deviate from the actual composition as mentioned before due to the limitation of Vegard’s law in determining the composition of NCs having strain. In the latter case, the EXAFS results obtained at the end of the reductive treatment showed higher coordination numbers of 2.66 ± 0.5 in the Pd–Cu_1_ first shell and 1.04 ± 0.63 in the Pd–Cu_2_ s shell for Pd–Cu compared to those obtained for the Pt–Cu alloy (CN Pt–Cu_1_ = 1.52 ± 0.6, CN Pt–Cu_2_ = 0.76 ± 0.3). This suggested that a greater number of Cu atoms are present around the Pd atoms after the reductive treatment.

Thus, the different extent of NC dealloying/migration/realloying process depending on the type of noble metal for these two systems could be explained considering the following considerations: (i) the slightly higher oxophilicity^62^ of Cu compared to Pd and Pt makes it a sacrificial element toward oxidation. Indeed, in the case of Pd–Cu NCs system, the formation of CuO was favored by the more negative Gibbs free energy of formation of CuO with respect to PdO (Δ*G*_CuO_ = −127 ± 4 kJ/mol and Δ*G*_PdO_ = −65 ± 8 kJ/mol, respectively).^63,64^ Then, the oxidation of Pd NCs was driven by the formation of the CuO–Pd interface due to the lower energy barrier for the penetration of oxygen in the Pd lattice at the CuO–Pd interface.^65^ For Pt–Cu NCs, it has been shown previously that the adsorption of oxygen onto non-supported Pt–Cu NCs could induce segregation of the 3d transition metal onto the NC surface due to the strong interaction with oxygen (heat of formation of oxide for Cu −150 kJ/mol and for Pt −50 kJ/mol).^19,63,66^ In particular, the exposure to oxygen causes outward diffusion of Cu and encapsulation of the particles by the formed Cu oxide layer due to the lower surface energy of CuO (<1 J/m^2^) compared to that of Pt (1.9 J/m^2^).^67^ Furthermore, due to the larger atomic radius of Pt compared to Cu and the presence of more strain in the Pt–Cu NCs, the dealloying process is more favored in this system compared to the Pd–Cu one, in which the formation of PdCuO mixed oxide was able to retain the oxidized Cu. (ii) After reduction, the heat of formation of the Pd–Cu solid solution bulk alloy is −14 kJ/mol,^68^ while that of the Pt–Cu solid solution is −11 kJ/mol.^68^ This indicates that the formation of these alloys is favored in both cases, with a slightly increased stability for the Pd–Cu alloy. However, the incomplete restoration of the initial Pt–Cu NC alloy compared to the Pd–Cu one occurred due to the partial segregation of Pt driven by minimization of strain energy (due to the difference in surface energies and atomic radius between Pt and Cu).^19,66,69^ A similar behavior was found in other Pt-based bimetallic alloys such as Pt–Cu, Pt–Ni, Pt–Fe, and Pt–Co.^66^ Additionally, the presence of a larger fraction of CuO_*x*_ species onto the support in the Pt–Cu system might result in a considerable fraction of Cu unavailable for alloying with Pt.

In the context of the studies carried out so far in our research group addressing bimetallic alloys of noble and non-noble metals,^25−27^ we speculate that similar synergies are expected to operate for nanoalloys containing other noble metals, depending on the actual compositions and phase structures. Specifically, the non-noble part acts like a sacrificial component that segregates from the alloy and prevents sintering of the noble metal. The newly formed species on the surface of the non-noble metal could have a potential role in the catalytic properties. However, the extent of this outcome needs to be further explored in future works to rationalize the effect on the catalytic properties of such materials for the desired reaction.

## Conclusions

In this work, we have studied the dynamics of structural transformations of Pd–Cu and Pt–Cu NCs supported on *γ-*Al_2_O_3_ while heating them under exposure to a sequence of oxidizing and reducing gas atmospheres. Specifically, the oxidizing treatment led to a different scenario depending on the type of noble metal used. PdCuO mixed oxide was found in the case of supported Pd–Cu alloy NCs, along with PdO and a small fraction of CuO_*x*_, while for Pt–Cu alloy NCs, a larger amount of CuO_*x*_ species migrated away from the Pt/PtO NCs on the support. The reducing treatment largely restored the Pd–Cu alloy NCs, and partially also the Pt–Cu ones, highlighting the different behavior depending on the type of noble metal and spatial distribution of CuO_*x*_.

We, therefore, concluded that the noble metal present in the bimetallic Cu-based alloy NCs has a strong influence on the dealloying/migrations/realloying processes occurring under typical heterogeneous catalytic reactions. Hence, the present work provides useful insights into the preparation of materials for catalysis. Indeed, the addition of nonprecious metal to Pd/Pt reduces the mobility of the active Pd/Pt metal sites onto the alumina support, stabilizing them against sintering into large clusters under reaction conditions. The implications of these findings on elucidating the transformations of supported noble metal–Cu alloyed NCs upon different activation methods are significant and motivate our ongoing investigations on the fine-tuning catalytic activity of these NCs in the CO oxidation reaction.

## Supporting Information Available

TGA data of as-prepared Pd–Cu, Pt–Cu, Cu, Pd, and Pt NCs; BF-TEM images of as-prepared Cu, Pd, and Pt NCs; HAADF-STEM images of alumina supported Pd–Cu, Pt–Cu, Cu, Pd, and Pt NCs after treatments; HRTEM images of alumina supported Pd–Cu NCs after oxidation; calculation of Pd_0.7_Cu_0.3_O NC size based on starting Pd–Cu NC size; CO DRIFT spectra on alumina supported Cu, Pd, and Pt NCs after treatments; XANES evolution spectra, concentration profiles of components at the Cu K-edge, and structural parameters from EXAFS data fitting of alumina supported Pd–Cu, Pt–Cu NCs during treatments (PDF)
