# Supplementary material for: Atmosphere-Induced Transient Structural Transformations of Pd–Cu and Pt–Cu Alloy Nanocrystals
Source: Chem Mater. 2021 Nov 10;33(22):8635–48. doi: 10.1021/acs.chemmater.1c02377 (PMC8619592; doi:10.1021/acs.chemmater.1c02377)
Supplement: Supplementary file 1 — cm1c02377_si_001.pdf [file cm1c02377_si_001.pdf]

Supporting Information

Atmosphere-Induced Transient Structural Transformations of Pd-Cu and Pt-Cu Alloy Nanocrystals

Lea Pasquale,[†,‡,*] Sharif Najafishirtari,[†,*,#] Rosaria Brescia,[§] Alice Scarpellini,[§] Cansunur Demirci,[†,‡] Massimo Colombo,[†] and Liberato Manna[†]

[†] Department of Nanochemistry, Istituto Italiano di Tecnologia, Via Morego 30, 16163 Genova, Italy

[‡] Dipartimento di Chimica e Chimica Industriale, Università degli Studi di Genova, Via Dodecaneso 31, 16146 Genova, Italy

[§] Electron Microscopy Facility, Istituto Italiano di Tecnologia, Via Morego 30, 16163 Genova, Italy

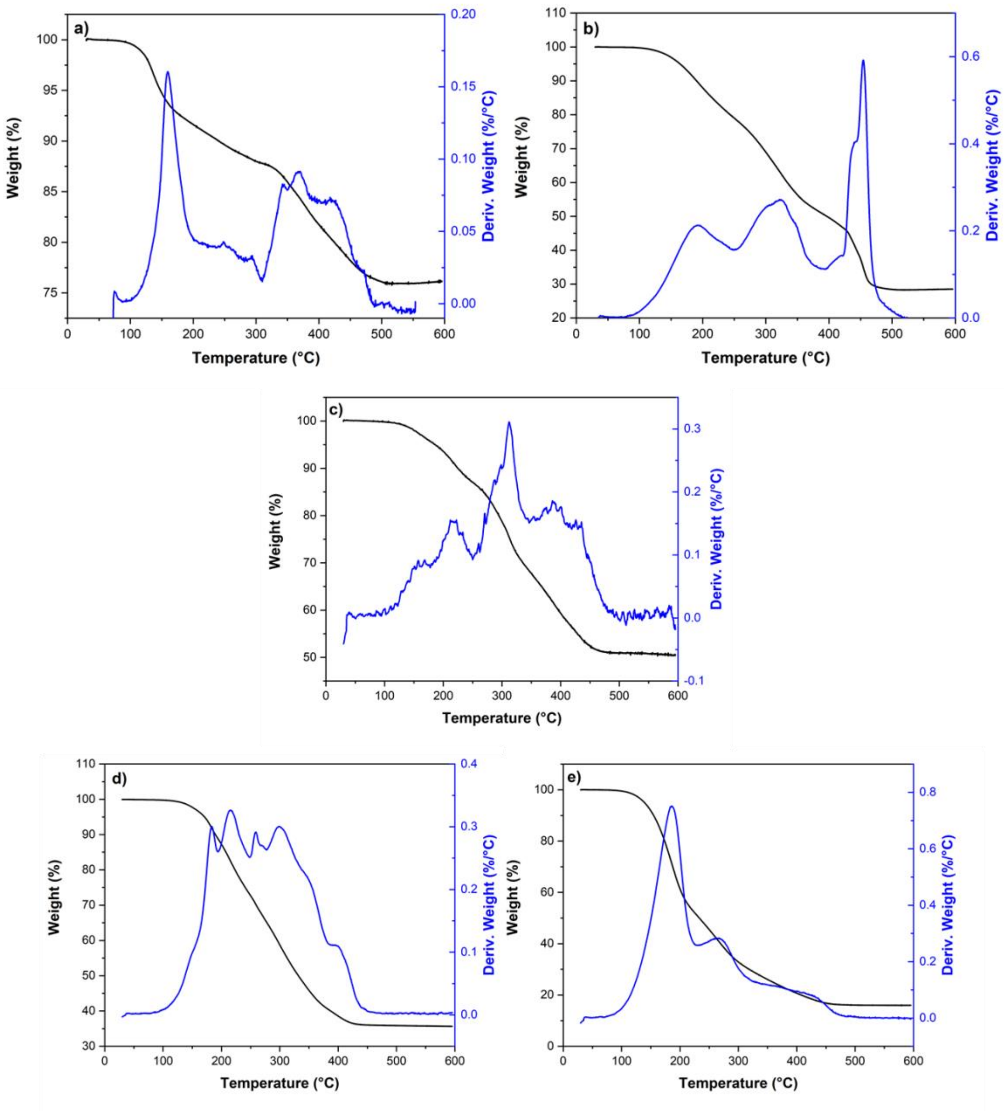

Figure S1. TGA of (a) Pd-Cu, (b) Pt-Cu (c) Cu, (d) Pd and (e) Pt NC colloidal solution under air flow. Weight is reported as a function of time.

The TGA was performed on a Q500 instrument from TA Instruments. After solvent evaporation, the sample (~3 mg of NCs) was equilibrated at 30 °C for 30 min, then heated to 600 ºC with a 10 °C min^{-1} heating rate under 50 mL min^{-1} of air flow. On the basis of the weight loss as a function of time, it can be seen that the thermal annealing at 450 °C in air was efficient for removing the organic surfactants from the bi- and monometallic NC surface.

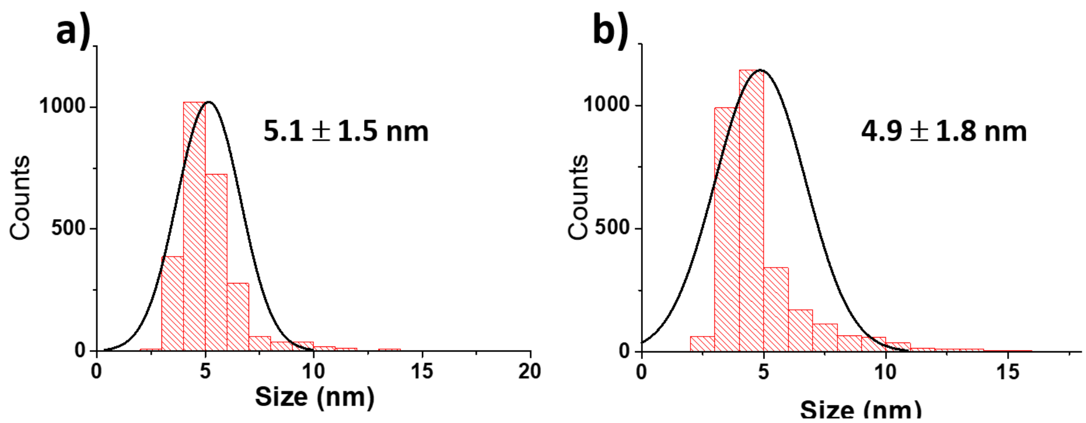

Figure S2. Size distribution histograms for (a) Pd-Cu NCs obtained by measuring 755 NCs and for (b) Pt-Cu NCs by measuring 1445 NCs.

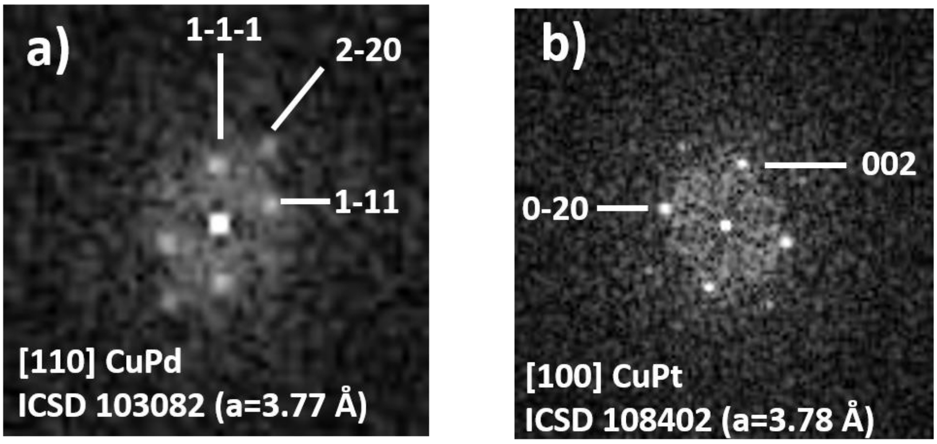

Figure S3. FFTs corresponding to (a) Figure 1c, matching with the [110] zone-axis projection of fcc CuPd (ICSD 103082) and (b) Figure 1g, matching with the [100] zone-axis projection of fcc CuPt (ICSD 108402).

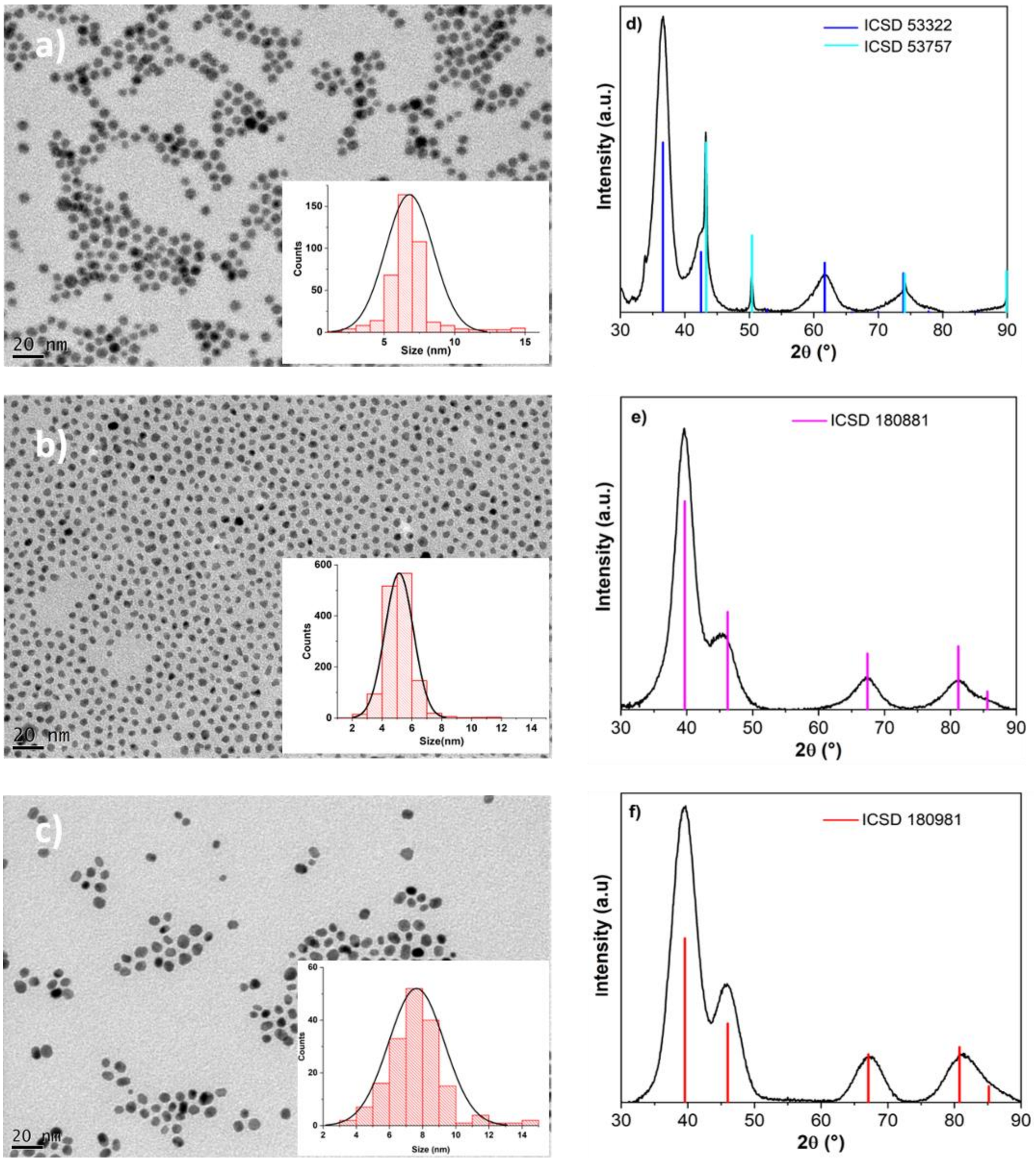

Figure S4. BF-TEM images of the as-synthesized (a) Cu, (b) Pd, (c) Pt NCs, with the corresponding size distribution histograms displayed as insets, and (d, e, f) XRD patterns with the expected peak position reference for fcc Cu (cyan, ICSD 53757) and fcc Cu_2O_1 (blue, ICSD 53322), fcc Pd (magenta, ICSD 180881) and fcc Pt (red, ICSD 180981).

The NC size and shape of monometallic Cu, Pd and Pt NCs were analyzed by *BF-TEM* (Figure S2 a-c). The average size of Cu, Pd and Pt NCs were 6.8 ± 1.7 nm based on 405 particles, 5.2 ± 1.0 nm on 1375 particles, and 7.6 ± 1.7 nm on 174 particles, respectively. The XRD patterns collected from the NCs were in agreement with fcc-Cu_2O, fcc-Pd and fcc-Pt structures (Figure S2 d-f). As reported by Yin et al.,[1] the presence of sharper peaks indexed to the fcc of metal Cu in the pattern of Cu NCs can be related to NC size larger than 5 nm.[1] However, it cannot be explicitly concluded that the particle size is 5 nm as the XRD gives information about the crystallite or domain size rather than particle size.

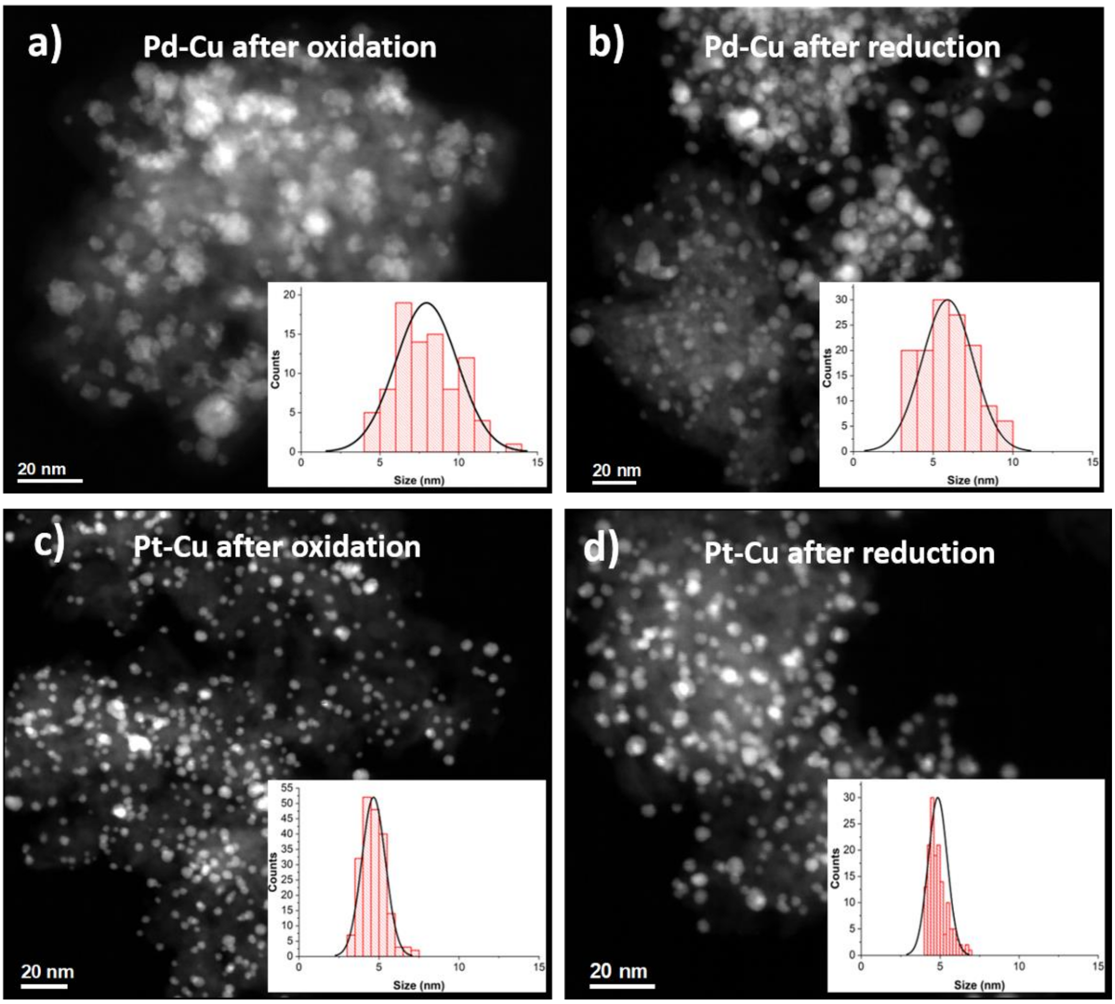

Figure S5. HAADF-STEM images of alumina supported (a, b) Pd-Cu and (c, d) Pt-Cu NCs after oxidizing (a, c) and reducing (b, d) treatments with the corresponding size distribution histograms displayed as insets.

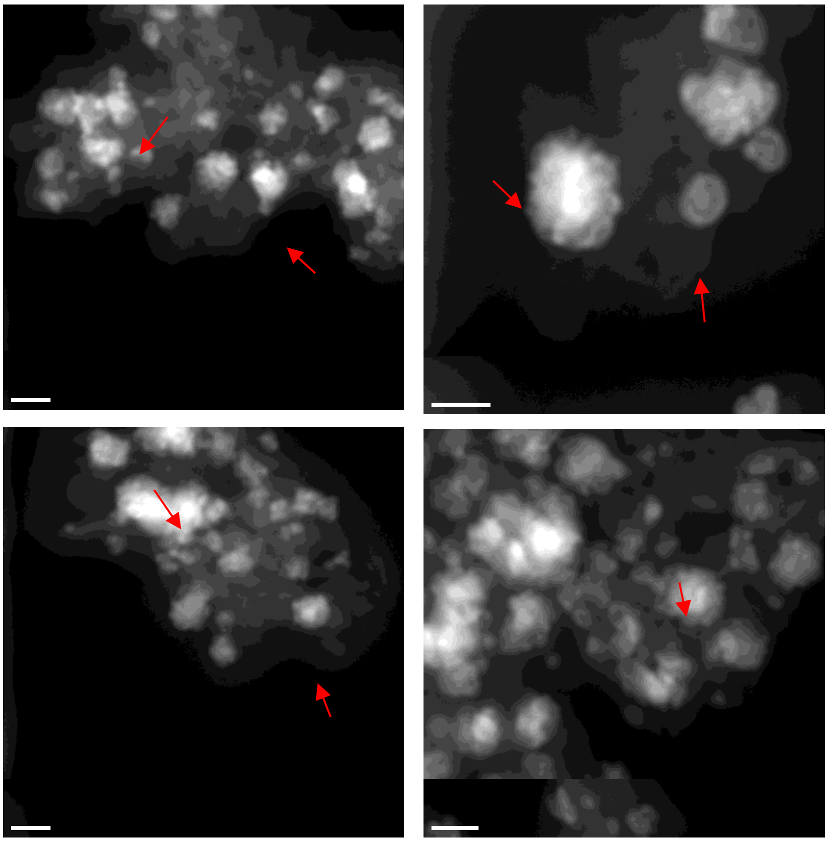

Figure S6. HAADF-STEM images of alumina supported Pd-Cu NCs after oxidizing treatment.

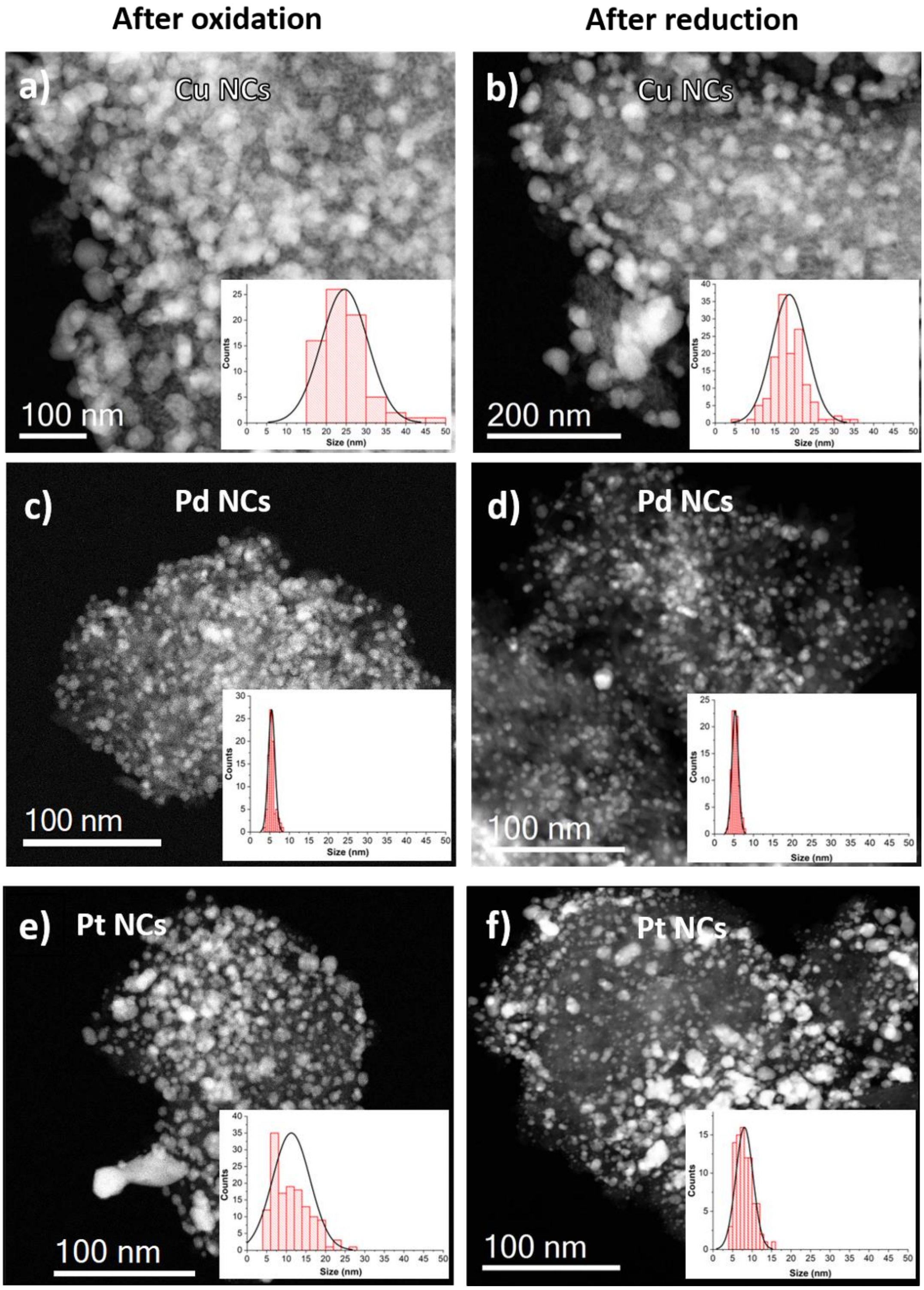

Figure S7. HAADF-STEM images of alumina supported (a, b) Cu, (c, d) Pd and (e, f) Pt NCs after oxidizing (a, c, e) and reducing (b, d, f) treatments with the corresponding size distribution histograms displayed as insets.

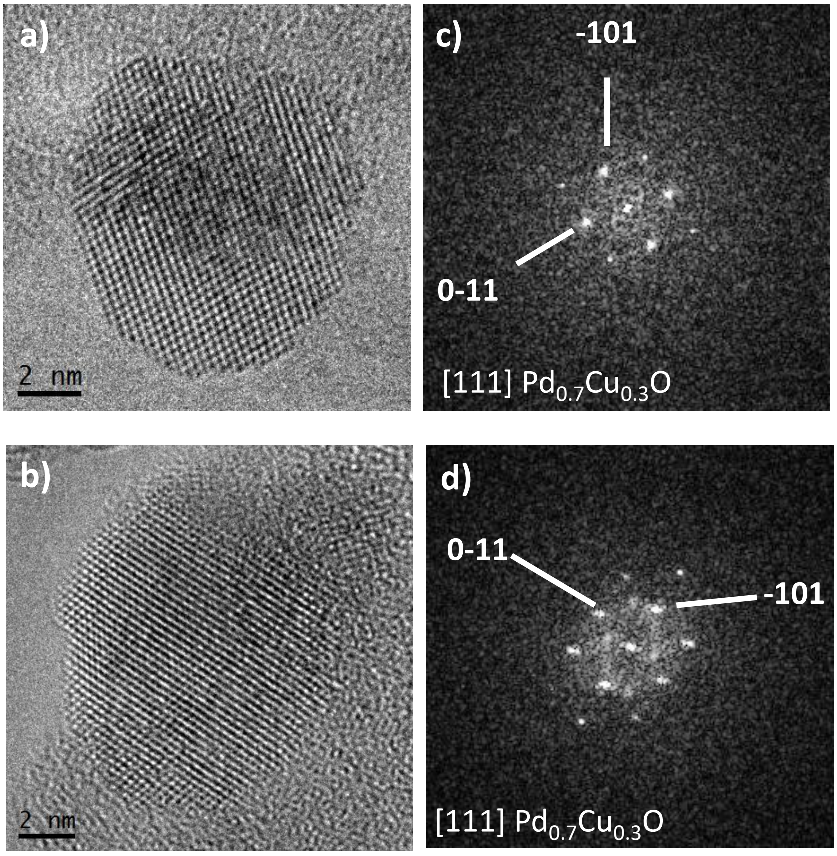

Figure S8. (a, b) HRTEM images of single-crystal Pd-Cu nanoparticles of alumina supported Pd-Cu NCs after oxidation with the corresponding (c, d) FFT patterns, matching with $Pd_{0.7}Cu_{0.3}O$.

Calculation of $Pd_{0.7}Cu_{0.3}O$ NC size based on starting Pd-Cu NC size

To estimate the enlargement of $Pd_{0.5}$-$Cu_{0.5}$ NC size ascribed to the formation of $Pd_{0.7}Cu_{0.3}O$ NCs, the following calculations are taken into account. First, considering the number of Pd atoms in the unit cell equal to 4, as well as the known unit cell volume of Pd-Cu (53.41 Å^3 in the Pd_1Cu_1 card ICSD 103082), the number of Pd atoms in the NC was calculated from the following expression:

$$N_{Pd\ atoms\ in\ PdCu\ NC} = \frac{N_{Pd\ atoms\ per\ unit\ cell}}{V_{PdCu\ unit\ cell}} V_{PdCu\ NC}$$

(Eq. S1)

where $V_{PdCu\ NC}$ is the volume calculated by the average of the NC size. The number of Cu atoms in the NC is equal to that of Pd being the atomic Pd:Cu ratio = 50:50. The expected volume $V_{Pd0.7Cu0.3O\ NC}$ of oxidized NC was calculated considering the unit cell volume equal to 47.77 Å^3 and the number of Cu atoms per unit cell of $Pd_{0.7}Cu_{0.3}O$ equal to 0.6 as follows, assuming that the NC has a spherical shape:

$$V_{Pd0.7Cu0.3O\ NC,Cu} = \frac{\mathrm{N}_{Cu\ atoms\ in\ Pd0.7Cu0.3O\ NC}}{\mathrm{N}_{Cu\ atoms\ per\ Pd0.7Cu0.3O\ unit\ cell}} V_{Pd0.7Cu0.3O\ unit\ cell}$$

(Eq. S2)

The unit cell volume of $Cu_{0.3}Pd_{0.7}O$ was calculated by modifying the tetragonal cell of PdO (ICSD 24692). The new cell has Cu and Pd ions with fractional occupancies (0.3 and 0.7, respectively) in the sites of Pd ions and parameters, a = b = 3.005 Å and c = 5.29 Å, calculated according to the plots in ref. 2 for $Cu_xPd_{1-x}O$, with x = 0.3.

The size of NC is then given by:

$$d_{Pd0.7Cu0.3O\ NC,Cu} = 2r_{Pd0.7Cu0.3O\ NC,Cu} = 2\sqrt[3]{\frac{3V_{Pd0.7Cu0.3O,Cu}}{4\pi}}$$

(Eq. S3)

where d and r are the diameter and radius of NC, respectively.

From the experimental data, the average size of the starting $Pd_{0.5}$-$Cu_{0.5}$ NC is 4.9 ± 1.8 nm while that calculated for oxidized NC with the Equations (S3) is 7.0 nm, value in agreement with that observed in the HAADF-STEM images (7.8 ± 2.0 nm). This means that the growth of NC size is 43% referred to the starting diameter.

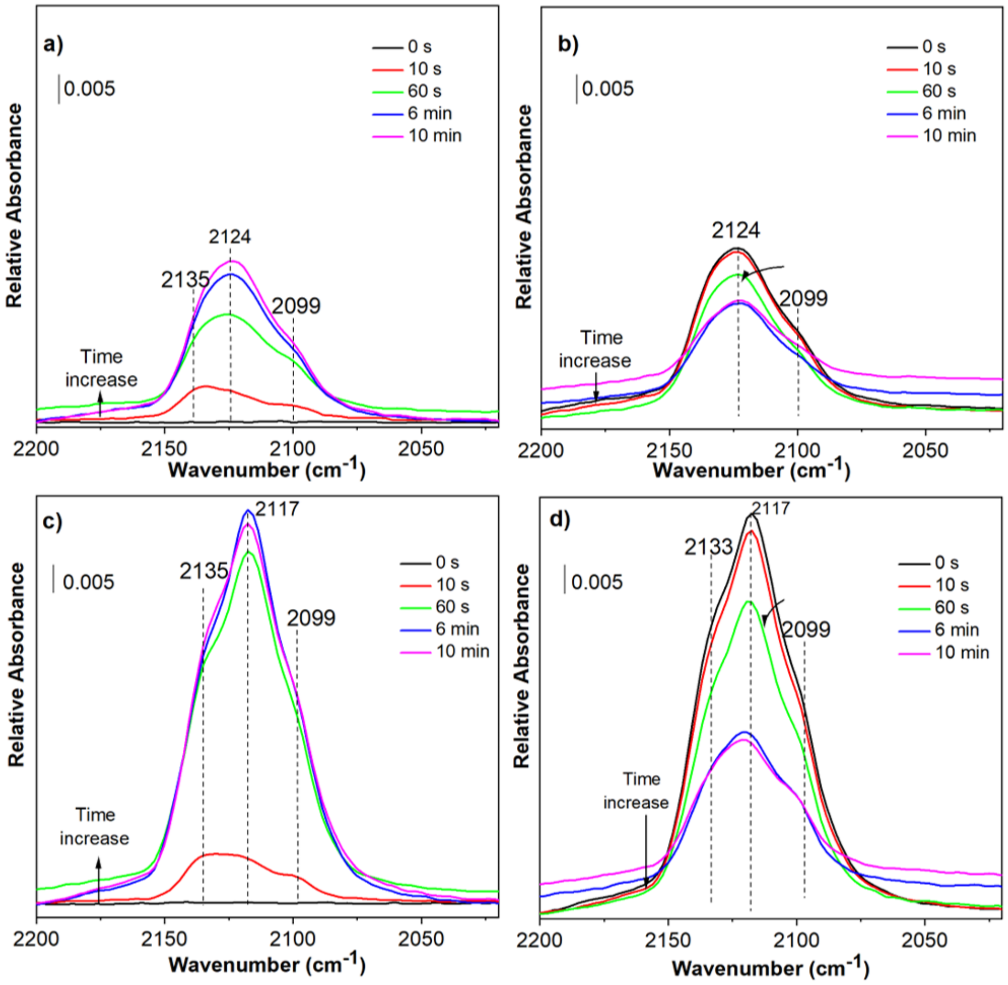

Figure S9. DRIFT spectra in the carbonyl region recorded during the adsorption (a, c) and desorption (b, d) of CO at room temperature on alumina supported Cu NCs after the (a, b) oxidizing and (c, d) reduction treatments.

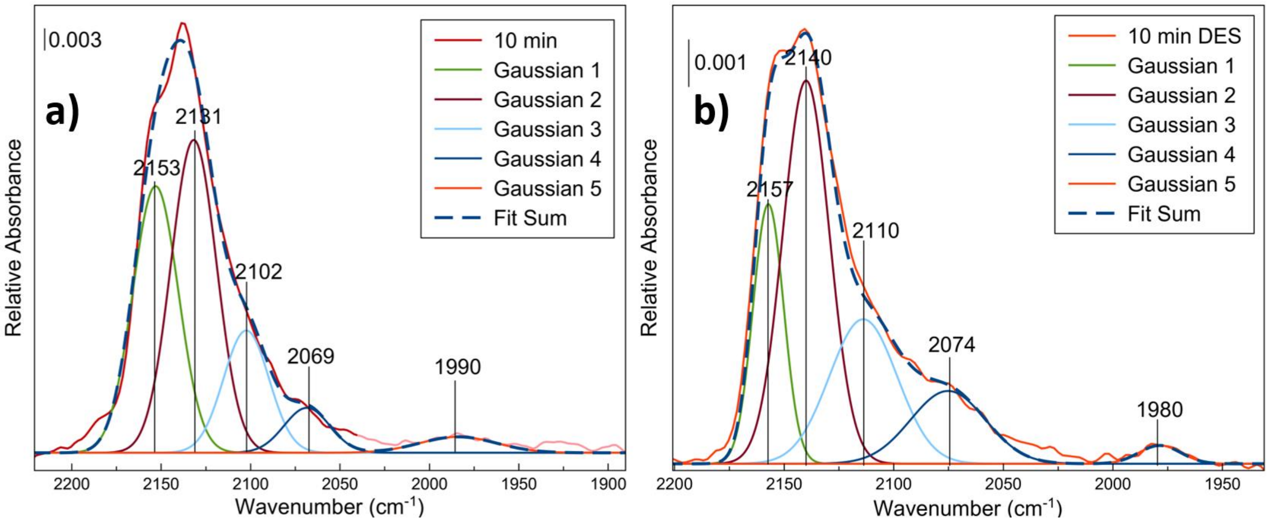

Figure S10. Deconvolution of DRIFT spectra recorded at 10-min of CO (a) adsorption and (b) desorption on Pd-Cu alloy NCs supported on Al_2O_3 after oxidation.

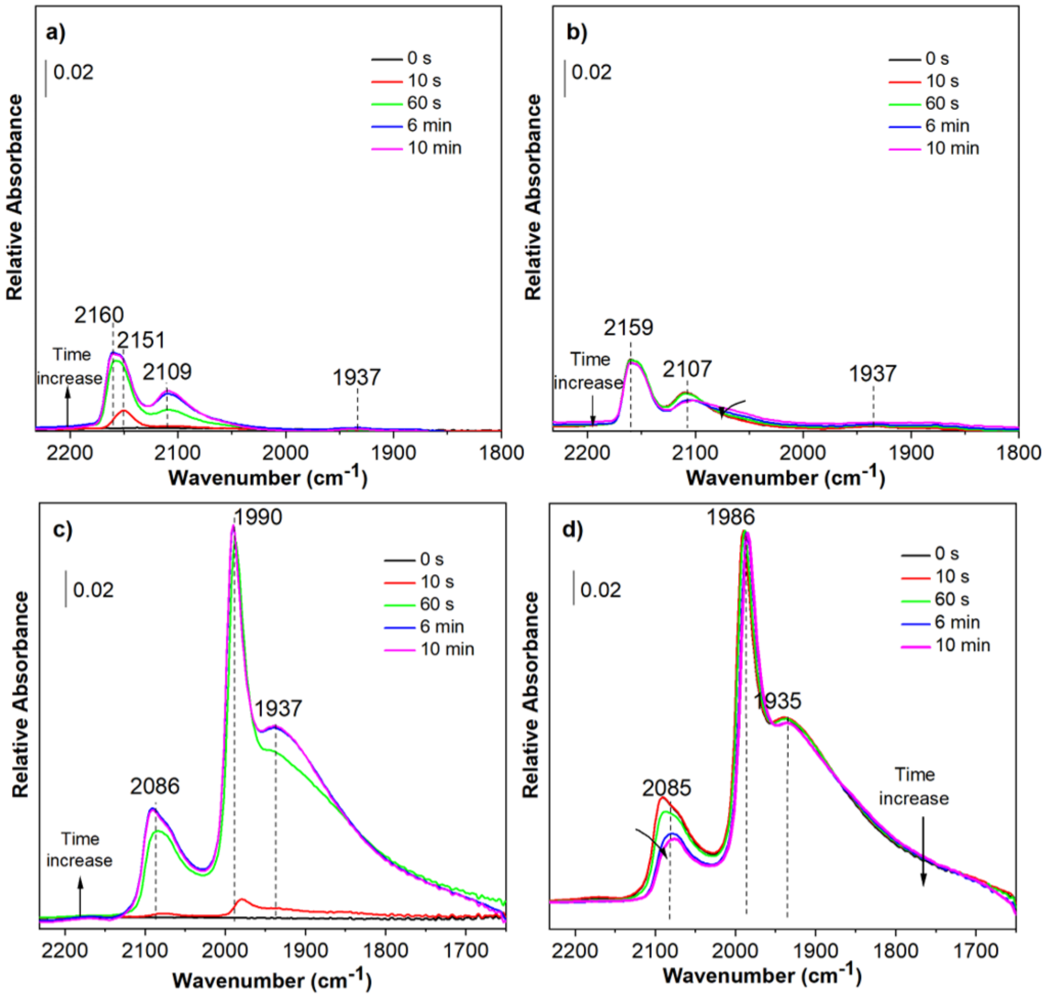

Figure S11. DRIFT spectra in the carbonyl region recorded during adsorption (a, c) and desorption (b, d) of CO at room temperature on alumina supported Pd NCs after (a, b) oxidizing and (c, d) reduction treatments.

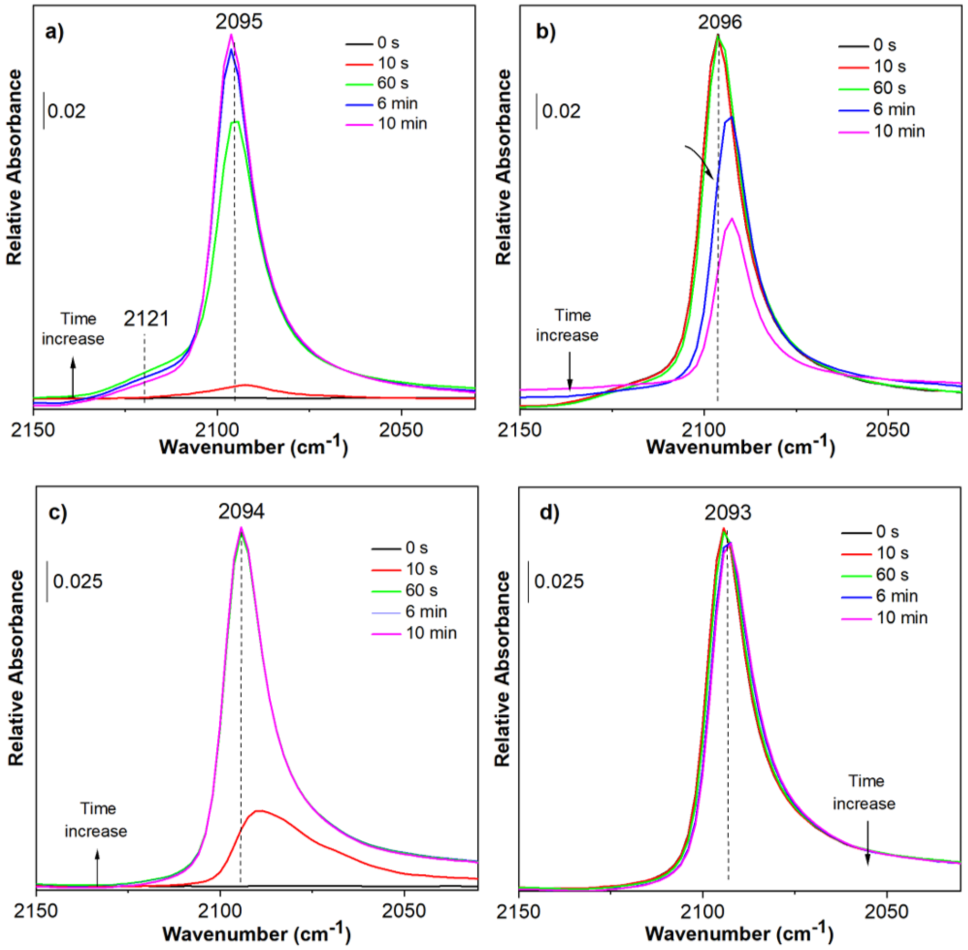

Figure S12. DRIFT spectra in the carbonyl region recorded during adsorption (a, c) and desorption (b, d) of CO at room temperature on alumina supported Pt NCs after (a, b) oxidizing and (c, d) reduction treatments.

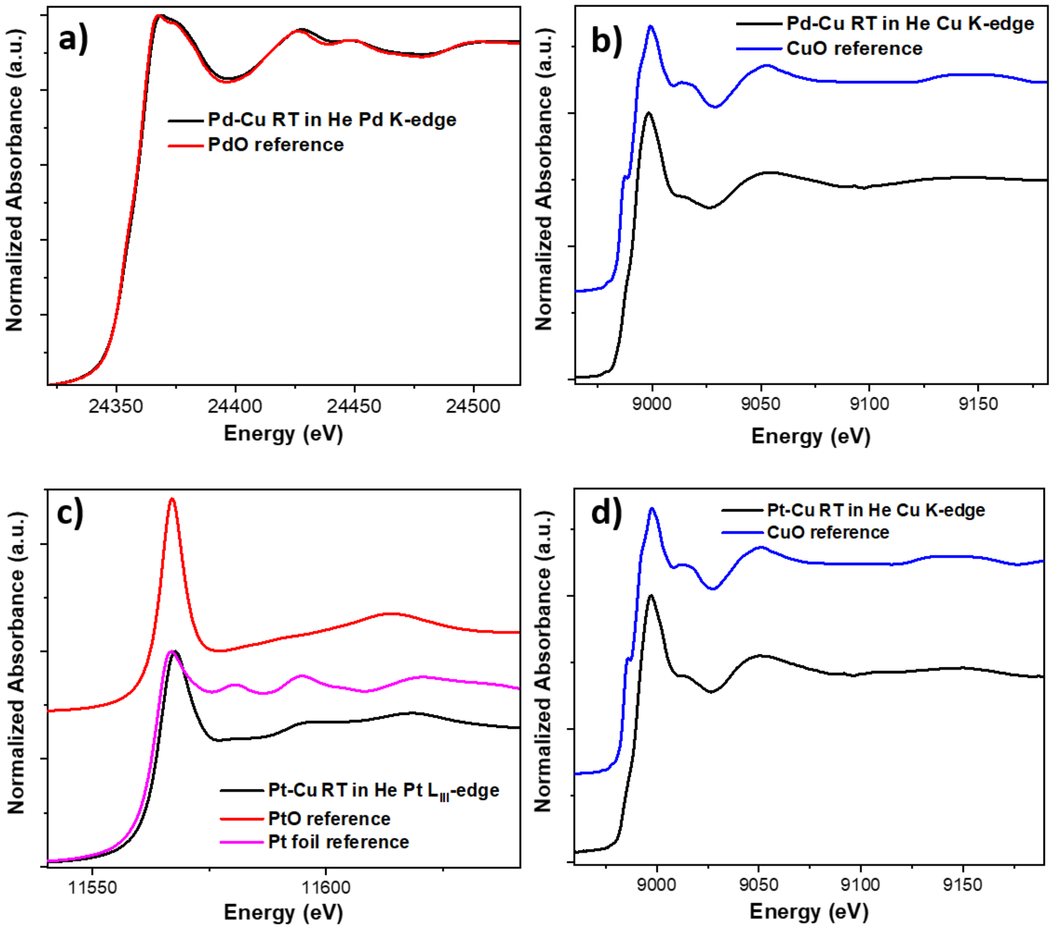

Figure S13. XANES spectra of alumina supported Pd-Cu (a, b) and Pt-Cu (c, d) NCs collected at RT in He at (a) Pd K-edge and (b) Cu K-edge along with the XANES spectra of PdO and CuO (collected at the SuperXAS beamline of the SLS) references and at (c) Pt L_{III}-edge and (d) Cu K-edge along with the XANES spectra of Pt foil reference, and PtO and CuO reference spectra collected at SuperXAS beamline of the SLS.

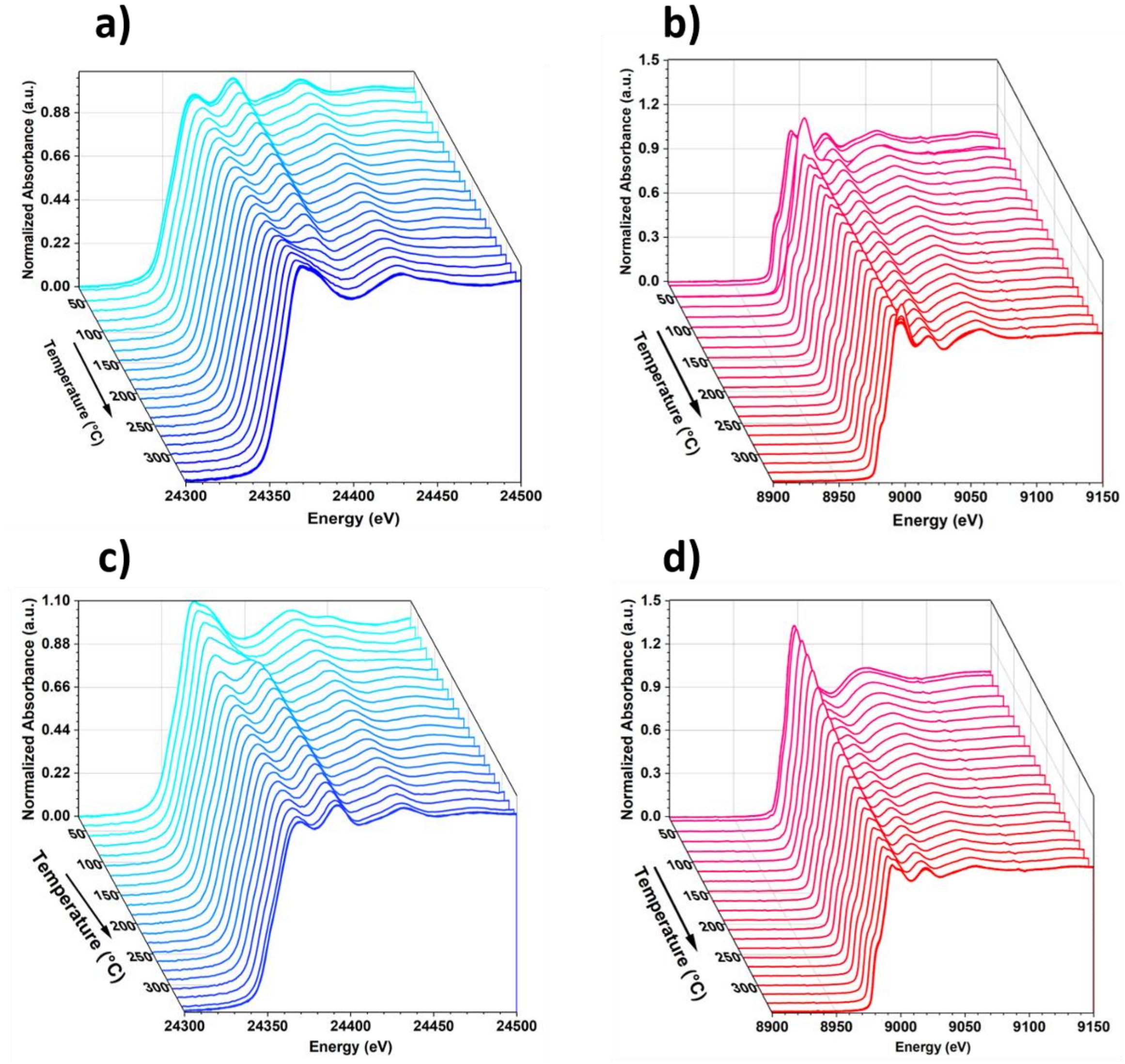

Figure S14. In situ XANES evolution of the alumina supported Pd-Cu NCs (a, c) at the Pd K-edge and (b, d) at the Cu K-edge during (a, b) the oxidizing and (c, d) the reduction treatments.

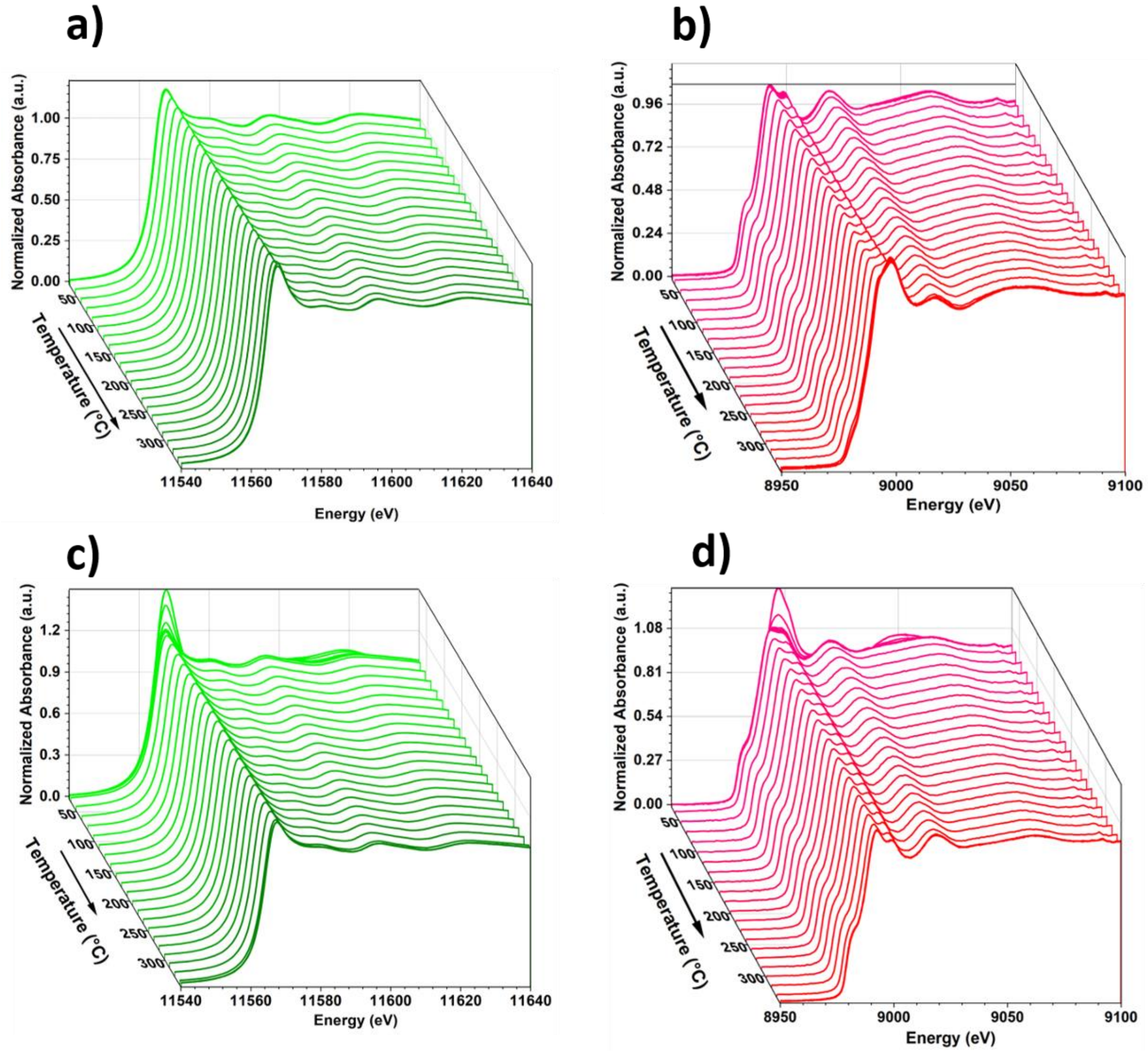

Figure S15. In situ XANES evolution of the alumina supported Pt-Cu NCs (a, c) at the Pt L_{III}-edge and (b, d) at the Cu K-edge during (a, b) the oxidizing and (c, d) the reduction treatments.

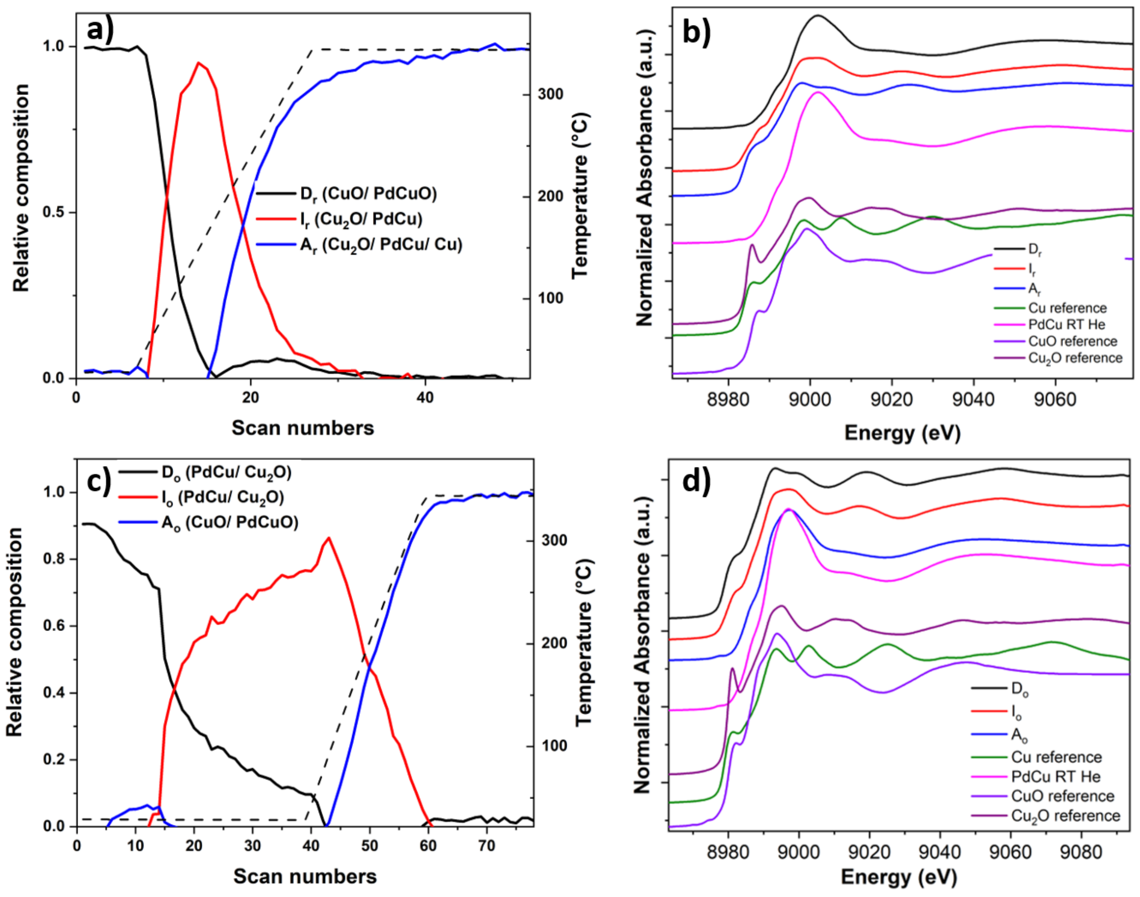

Figure S16. Concentration profiles of components as a function of the temperature during reduction (a) and during oxidation (c) at the Cu K-edge. XANES spectra of the species formed during reduction (b) and during oxidation (d) for the alumina supported Pd-Cu NCs along with the XANES spectra of Cu foil reference, CuO and Cu_2O reference spectra collected at the SuperXAS beamline of the SLS, and Pd-Cu NCs spectrum collected at room temperature in He.

During reduction at the Cu K-edge of the Pd-Cu NCs, the concentration profile as a function of temperature evidenced a two-step reduction process (Figure S16a) in which the component D_r started to be significantly reduced into the intermediate component I_r at T~50 °C and then the formation of the component A_r started to be significant at T>150 °C. The comparison between the pure component spectra with the reference ones (Figure S16b) suggested that the D_r consists of a fingerprint of CuO, the I_r of Cu_2O one and A_r to the metallic Cu structure. The best fit of the FT of the EXAFS spectra (Table S2) for the component D_r presented contributions of Cu-O and Cu-Cu coordination shells at 2.00 Å and at 2.97 Å, characteristic of CuO, and a lesser extent of PdCuO mixed oxide. The best fit of I_r component was based on the contribution of two species. Specifically, Cu-O and Cu-Cu shells at 1.94 Å and 3.00 Å related to Cu_2O and Cu-Cu one at 2.65 Å referred to Pd-Cu were found. Finally, the A_r component was mainly characterized by a single Cu-Cu coordination shell at 2.48 Å related to metallic Cu with a minor contribution of Cu-Cu shell at 2.58 Å of Pd-Cu species and Cu-O shell at 1.86 Å for Cu_2O.

When the reduced NCs were subjected to an oxidative environment at the Cu K-edge, three components named D_o (descending), I_o (intermediate) and A_o (ascending) were identified and presented as a function of the temperature in Figure S16c, d along with the corresponding spectra. Initially, already a room temperature under the

exposure to the oxygen, the decrease of the constituent D_0 proceed parallel with the appearance of constituent I_0; as soon as the temperature started to increase in the heating ramp, the component A_0 progressively increased until reaching the complete oxidation at 350 °C. The comparison between the XANES spectra with the reference ones (Figure S16d) revealed the formation of the intermediate Cu_2O as component I_0. This was confirmed from its absorption edge lying in between those of the constituents D_0 and A_0, related to the Cu^{+2} and CuO species, respectively. The EXAFS signal and FT of the components D_0 and I_0 required two contributions for a better fit (Table S2), one from Cu and Pd atoms in an ordered CuPd alloy and one from O atoms in Cu_2O. In particular, the contribution of the first one decreased from D_0 to I_0 with the increase of the second one (coordination number (CN) of Cu-O equal to 0.49 for D_0 to 1.97 for I_0). Finally, the best fit for A_0 confirmed the formation of CuO with a minor extent of PdCuO.

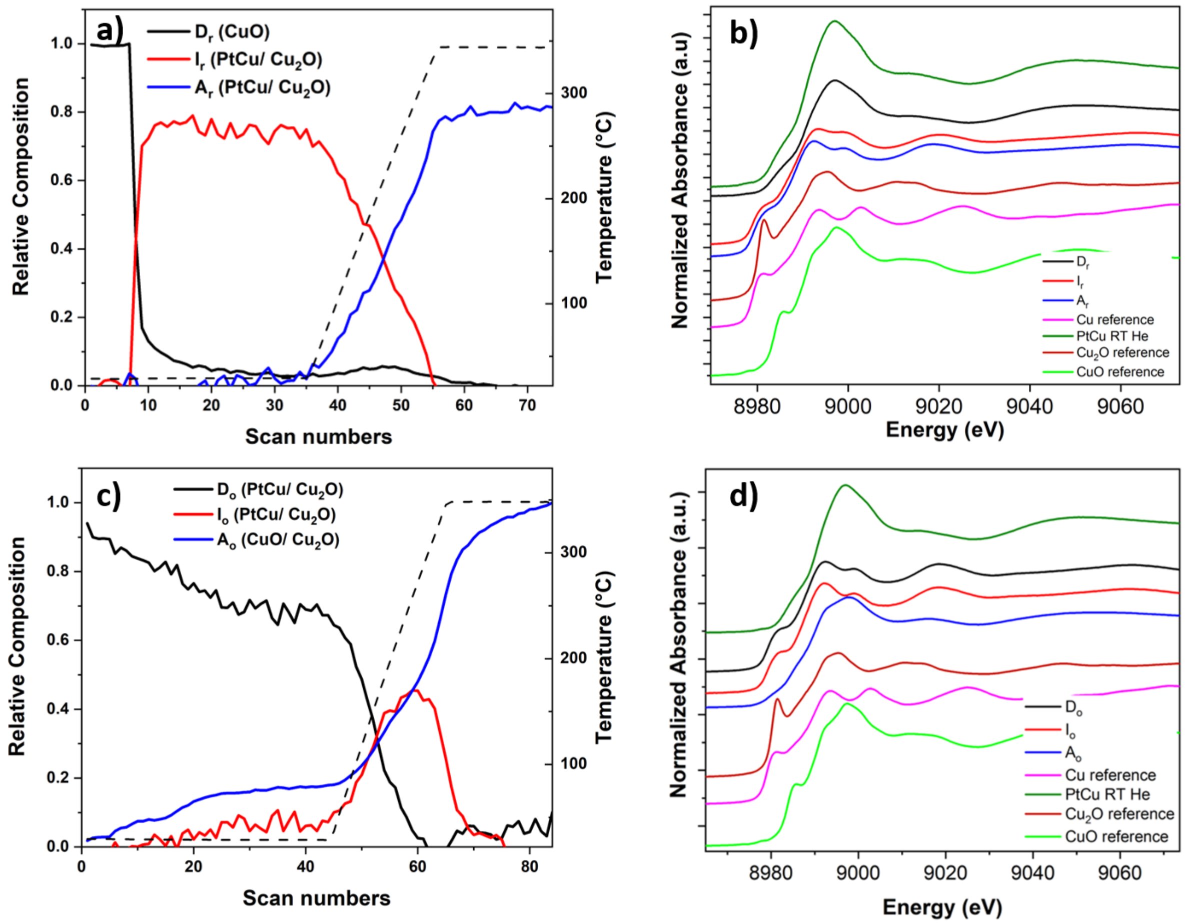

Figure S17. Concentration profiles of components as a function of the temperature during reduction (a) and during oxidation (c) at the Cu K-edge. XANES spectra of the species formed during reduction (b) and during oxidation (d) for the alumina supported Pt-Cu NCs along with the XANES spectra of Cu foil reference, CuO and Cu_2O reference spectra collected at SuperXAS beamline of the SLS, and Pt-Cu NCs spectrum collected at room temperature in He.

During reduction at the Cu K-edge of the Pt-Cu NCs, the spectrum of the descending D_r component (Figure S17b) was similar to that of CuO, while the second (intermediate component I_r) and the third (ascending component A_r) ones showed characteristic features similar to those of Cu_2O and metallic Cu,[3-4] respectively. The concentration dependence of these three components (Figure S17a) proved that first, D_r started to be reduced already at room temperature into I_r, and at the beginning of the heating ramp, A_r started to be significant. As suggested by the qualitative analysis of the XANES part, the structural parameter determined by the fitting of the FT (Table S4) confirmed the presence of CuO as starting species assigned to D_r. The intermediate I_r component showed a main peak in the R-space around 2.60 Å compatible with Cu-Cu fist neighbors in the fcc Pt-Cu structure. A minor contribution at 1.90 Å was related to oxygen neighbors around Cu absorbing atoms for Cu_2O. Finally, the A_r component was satisfactorily described by a Cu poor disordered alloy (CN Cu-Cu = 3.28 ± 0.6, CN Cu-Pt = 5.34 ± 0.7) with a minor contribution at 1.93 Å related to Cu_2O. The low coordination number of 0.33 found for this last species suggested the formation of small clusters. This result describing the final state at the end of the reduction was in line with those

obtained at the Pt L_{III}-edge, corresponding with the formation of a Pt-rich Pt-Cu alloy, corroborated by the SAED analysis.

During oxidation at the Cu K-edge, three components involved during the oxidative treatment at Cu K-edge (descending D_o, intermediate I_o and ascending A_o components) were found (Figure S17c). A decrease in the relative fraction of the component D_o was paralleled by the transient growth in the contribution of the Io and a minor fraction to A_o. Then, at the beginning of the heating ramp, I_o was progressively increased along with A_o until it disappeared as the temperature reached 350 °C (Figure S17c). The best fit of the FT EXAFS spectra for D_o and I_o (Table S4) showed scattering at 2.64 Å due to the Cu atoms in the first coordination shell and Pt atoms in the second one related to the formation of the fcc Pt-Cu alloy. These two components differed only in the Cu-Cu coordination number slightly higher for I_o. A small contribution at around 1.90 Å was observed for the fitted Cu-O shell related to Cu_2O. Finally, the complete oxidation of Cu was evident for the component A_o from the presence of the Cu-O shell at 1.95 Å related to CuO with an increased intensity of the Cu-O interaction at 2.0 Å for Cu_2O compared to what found for D_o and I_o.

Table S1: Structural parameters obtained from the fitting of EXAFS at the Pd K-edge for the alumina supported Pd-Cu NCs (k range for the Fourier transform 2.5-12.4 Å^{-1}, the R-range for the fitting was 1-3.5/4 Å).

	Scattering Path	*CN*[1]	*R (Å)*	*σ^2(Å^2)*	*R-factor*
			PdCu		
**As-synthetized Pd-Cu NCs**	Pd-Pd1	5.30*	2.703 ± 0.009	0.009 ± 0.001	0.011
	Pd-Cu1	5.68*	2.625 ± 0.017	0.014 ± 0.002	
	Pd-Pd2	1.38*	3.806 ± 0.009	0.009 ± 0.001	
	Pd-Cu2	1.04*	3.728 ± 0.017	0.014 ± 0.002	

	Scattering Path	*CN*	*R (Å)*	*σ^2 (Å^2)*	*R-factor*
			PdO		
**Red. D_r**	Pd-O1	2.80*	2.026*	0.003 ± 0.001	0.016
	Pd-Pd1	1.86*	3.059 ± 0.006	0.006 ± 0.001	
			PdCuO		
	Pd-O1	0.68*	2.026*	0.003 ± 0.001	
	Pd-Cu1	1.69*	3.004 ± 0.031	0.015*	
	Pd-Pd1	1.34*	3.059 ± 0.006	0.006 ± 0.001	

	Scattering Path	*CN*	*R (Å)*	*σ^2(Å^2)*	*R-factor*
			PdCu		
**Red. A_r**	Pd-Pd1	7.30 ± 0.47	2.706 ± 0.006	0.011 ± 0.001	0.013
	Pd-Cu1	2.66 ± 0.47	2.639 ± 0.014	0.014 ± 0.003	
	Pd-Pd2	2.71 ± 0.63	3.809 ± 0.006	0.011 ± 0.001	
	Pd-Cu2	1.04 ± 0.63	3.742 ± 0.014	0.014 ± 0.003	

	Scattering Path	*CN*	*R (Å)*	*σ^2(Å^2)*	*R-factor*
			PdCu		
**Oxi. D_o**	Pd-Pd1	6.53 ± 0.56	2.717 ± 0.005	0.007 ± 0.001	0.017
	Pd-Cu1	3.43 ± 0.56	2.667 ± 0.013	0.011 ± 0.003	
	Pd-Pd2	1.94 ± 0.69	3.820 ± 0.005	0.007 ± 0.001	
	Pd-Cu2	1.04*	3.770 ± 0.013	0.011 ± 0.003	

	Scattering Path	*CN*	*R (Å)*	*σ^2(Å^2)*	*R-factor*
			PdO		
**Oxi. A_o**	Pd-O1	2.80*	2.026*	0.005 ± 0.001	0.023
	Pd-Pd1	3.08*	3.061 ± 0.017	0.012 ± 0.005	
			PdCuO		
	Pd-O1	0.44*	2.026*	0.005 ± 0.001	
	Pd-Cu1	0.67*	2.954 ± 0.081	0.012*	
	Pd-Pd1	0.15*	3.057 ± 0.011	0.012 ± 0.005	

	Scattering Path	*CN*	*R (Å)*	*σ^2(Å^2)*	*R-factor*
			PdO		
**RT He**	Pd-O1	2.80*	2.026*	0.003 ± 0.001	0.017
	Pd-Pd1	7.89*	3.058*	0.007 ± 0.001	
			PdCuO		
	Pd-O1	0.77*	2.027*	0.003 ± 0.001	
	Pd-Cu1	1.56*	3.027 ± 0.080	0.016*	
	Pd-Pd1	4.25*	3.059 ± 0.007	0.007 ± 0.001	

1 The calculation of CN at each shell was calculated as the product of NS_o^2.

*Parameters were fixed in the fitting and obtained as the product of N of the shell from the model and adjusted S_o^2 for the reference Pd.

Table S2: Structural parameters obtained from the fitting of EXAFS at the Cu K-edge for the alumina supported Pd-Cu NCs (k range for the Fourier transform 2.5-12.4 $Å^{-1}$, the R-range for the fitting was 1-3/4 Å).

	Scattering Path	*CN*	*R (Å)*	*σ²(Å²)*	*R-factor*
**As-synthetized Pd-Cu NCs**	**PdCu**				0.013
	Cu-Cu1	3.11*	2.663 ± 0.079	0.010*	
	Cu-Pd1	3.11*	2.654 ± 0.010	0.018 ± 0.002	
	**Cu_2O**				
	Cu-O1	0.36	1.962± 0.006	0.010± 0.002	
	Cu-Cu1	1.08	2.836± 0.079	0.018 ± 0.002	

	Scattering Path	*CN*	*R (Å)*	*σ² (Å²)*	*R-factor*
**Red. D_r**	**CuO**				0.010
	Cu-O1	1.15 ± 0.1	2.005 ± 0.010	0.003**	
	Cu-Cu1	2.31± 0.1	2.965 ± 0.024	0.007**	
	**PdCuO**				
	Cu-O1	1.26 ± 0.2	1.902 ± 0.010	0.003**	
	Cu-Cu1	0.95*	3.000 ± 0.024	0.007**	
	Cu-Pd1	0.32*	3.018 ± 0.056	0.012**	

	Scattering Path	*CN*	*R (Å)*	*σ² (Å²)*	*R-factor*
**Red. I_r**	**Cu_2O**				0.008
	Cu-O1	2.29 ± 0.4	1.944 ± 0.018	0.013 ± 0.003	
	Cu-Cu1	6.88 ± 1.3	3.001 ± 0.020	0.027 ± 0.003	
	**PdCu**				
	Cu-Cu1	2.26 ± 0.4	2.651 ± 0.020	0.027 ± 0.003	
	Cu-Pd1	2.26 ± 0.4	2.657 ± 0.016	0.013 ± 0.002	

	Scattering Path	*CN*	*R (Å)*	*σ² (Å²)*	*R-factor*
**Red. A_r**	**Cu_2O**				0.020
	Cu-O1	0.20*	1.845 ± 0.028	0.003 ± 0.004	
	Cu-Cu1	0.60*	3.013 ± 0.012	0.017 ± 0.001	
	**PdCu**				
	Cu-Cu1	1.58*	2.586 ± 0.043	0.017 ± 0.011	
	Cu-Pd1	2.58*	2.585 ± 0.056	0.015 ± 0.003	
	**Cu**				
	Cu-Cu1	2.58*	2.487 ± 0.043	0.017 ± 0.011	

	Scattering Path	*CN*	*R (Å)*	*σ² (Å²)*	*R-factor*
**Oxi. D_o**	**Cu_2O**				0.018
	Cu-O1	0.49*	1.877 ± 0.022	0.004 ± 0.003	
	**PdCu**				
	Cu-Cu1	3.43*	2.538 ± 0.013	0.009 ± 0.001	
	Cu-Pd1	3.43*	2.595 ± 0.019	0.013 ± 0.001	

	Scattering Path	*CN*	*R (Å)*	*σ² (Å²)*	*R-factor*
**Oxi. I_o**	**Cu_2O**				0.012
	Cu-O1	1.97*	1.906 ± 0.015	0.009 ± 0.003	
	Cu-Cu1	5.90*	3.044 ± 0.033	0.029 ± 0.004	
	**PdCu**				
	Cu-Cu1	2.94*	2.694 ± 0.033	0.029 ± 0.004	

	Cu-Pd1	2.94*	2.670 ± 0.016	0.012 ± 0.002	

	Scattering Path	*CN*	*R (Å)*	*σ² (Å²)*	*R-factor*
**Oxi. A₀**		***CuO***			0.010
	Cu-O1	1.02 ± 0.1	1.977 ± 0.005	0.003 ± 0.001	
	Cu-Cu1	2.05*	2.975 ± 0.018	0.040 ± 0.010	
		PdCuO			
	Cu-O1	1.25 ± 0.2	1.894 ± 0.005	0.003 ± 0.001	
	Cu-Cu1	0.94*	3.010 ± 0.018	0.040 ± 0.010	
	Cu-Pd1	0.31*	2.997 ± 0.020	0.017 ± 0.020	

	Scattering Path	*CN*	*R (Å)*	*σ² (Å²)*	*R-factor*
**RT He**		***CuO***			0.010
	Cu-O1	1.15 ± 0.1	2.008 ± 0.025	0.010**	
	Cu-Cu1	2.30 ± 0.1	2.978 ± 0.023	0.065**	
		PdCuO			
	Cu-O1	1.28 ± 0.1	1.905 ± 0.025	0.010**	
	Cu-Cu1	0.96 ± 0.1	3.012 ± 0.023	0.065**	
	Cu-Pd1	0.32 ± 0.1	3.016 ± 0.032	0.010**	

*Parameters were fixed in the fitting and obtained as the product of N of the shell from the model and adjusted S_o^2 for the reference Cu.

** Parameters fixed for the fitting.

Table S3: Structural parameters obtained from the fitting of EXAFS at the Pt L_{III}-edge for the alumina supported Pt-Cu NCs (k range for the Fourier transform 2.5-12.4 $Å^{-1}$, the R-range for the fitting was 1-3.5/4 Å).

	Scattering Path	*CN*	*R (Å)*	*σ²(Å²)*	*R-factor*
**As-synthetized Pt-Cu NCs**			***PtCu***		0.027
	Pt-Pt1	6.31 ± 0.8	2.706 ± 0.004	0.006 ± 0.0004	
	Pt-Cu1	2.77 ± 0.7	2.689 ± 0.010	0.009 ± 0.002	
	Pt-Pt2	3.16 ± 0.3	3.813 ± 0.004	0.006 ± 0.0004	
	Pt-Cu2	1.39 ± 0.4	3.796 ± 0.010	0.009 ± 0.002	

	Scattering Path	*CN*	*R (Å)*	*σ²(Å²)*	*R-factor*
**Red. D_r**			***PtO***		0.029
	Pt-O1	1.79 ± 0.2	2.008 ± 0.010	0.003 ± 0.001	
	Pt-Pt1	1.79 ± 0.2	3.070 ± 0.015	0.005 ± 0.001	
			Pt		
	Pt-Pt1	4.24 ± 0.6	2.762 ± 0.015	0.005 ± 0.001	

	Scattering Path	*CN*	*R (Å)*	*σ²(Å²)*	*R-factor*
**Red. A_r**			PtCu		0.023
	Pt-Pt1	8.00 ± 0.6	2.747 ± 0.005	0.008 ± 0.0004	
	Pt-Cu1	1.52 ± 0.6	2.665 ± 0.013	0.010 ± 0.003	
	Pt-Pt2	4.00 ± 0.3	3.852 ± 0.005	0.008 ± 0.0004	
	Pt-Cu2	0.76 ± 0.3	3.771 ± 0.013	0.010 ± 0.003	

	Scattering Path	*CN*	*R (Å)*	*σ²(Å²)*	*R-factor*
**Oxi. D_o**			***PtCu***		0.017
	Pt-Pt1	6.96 ± 0.8	2.721 ± 0.004	0.006 ± 0.0004	
	Pt-Cu1	2.83 ± 0.7	2.680 ± 0.011	0.008 ± 0.002	
	Pt-Pt2	3.48 ± 0.4	3.827 ± 0.004	0.006 ± 0.0004	
	Pt-Cu2	1.42 ± 0.4	3.787 ± 0.011	0.008 ± 0.002	

	Scattering Path	*CN*	*R (Å)*	*σ²(Å²)*	*R-factor*
**Oxi. A_o**			***Pt***		0.027
	Pt-Pt1	8.90 ± 0.7	2.734 ± 0.006	0.012 ± 0.001	
			PtO		
	Pt-O1	0.36 ± 0.1	2.068 ± 0.035	0.013 ± 0.002	
	Pt-Pt2	0.36 ± 0.1	3.225 ± 0.006	0.012 ± 0.001	

	Scattering Path	*CN*	*R (Å)*	*σ²(Å²)*	*R-factor*
**RT He**			***Pt***		0.021
	Pt-Pt1	3.44 ± 0.4	2.764 ± 0.004	0.005 ± 0.0004	
			PtO		
	Pt-O2	2.17*	2.010 ± 0.008	0.004 ± 0.001	
	Pt-Pt1	0.81*	3.082 ± 0.019	0.005 ± 0.0004	
	Pt-Pt2	1.63*	3.470 ± 0.019	0.005 ± 0.0004	

*Parameters were fixed in the fitting and obtained as the product of N of the shell from the model and adjusted S_o^2 for the reference Pt.

Table S4: Structural parameters obtained from the fitting of EXAFS at the Cu K-edge for the alumina supported Pt-Cu NCs (k range for the Fourier transform 2.5-12.4 Å^{-1}, the R-range for the fitting was 1-3.5/4 Å).

	Scattering Path	*CN*	*R (Å)*	*σ^2(Å^2)*	*R-factor*
**As-synthetized Pt-Cu NCs**			***CuO***		0.022
	Cu-O1	2.42 ± 0.4	1.993 ± 0.01	0.010 ± 0.002	
	Cu-Cu1	4.85 ± 0.7	2.880 ± 0.01	0.018 ± 0.002	
			PtCu		
	Cu-Cu1	1.64*	2.670 ± 0.01	0.018 ± 0.002	
	Cu-Pt1	1.64*	2.670 ± 0.01	0.010 ± 0.005	

	Scattering Path	*CN*	*R (Å)*	*σ^2(Å^2)*	*R-factor*
**Red. D_r**			***CuO***		0.021
	Cu-O1	2.77 ± 0.4	1.952 ± 0.012	0.003 ± 0.002	
	Cu-Cu1	5.54 ± 0.7	2.936 ± 0.023	0.020 ± 0.003	

	Scattering Path	*CN*	*R (Å)*	*σ^2(Å^2)*	*R-factor*
**Red. I_r**			***Cu_2O***		0.006
	Cu-O1	0.69 ± 0.2	1.901 ± 0.03	0.009 ± 0.013	
	Cu-Cu1	2.07 ± 0.7	2.950 ± 0.03	0.014 ± 0.004	
			PtCu		
	Cu-Cu1	4.58 ± 0.5	2.607 ± 0.03	0.014 ± 0.004	
	Cu-Pt1	4.00 ± 0.6	2.574 ± 0.10	0.013 ± 0.100	

	Scattering Path	*CN*	*R (Å)*	*σ^2(Å^2)*	*R-factor*
**Red. A_r**			***Cu_2O***		0.009
	Cu-O1	0.33 ± 0.2	1.927 ± 0.05	0.014 ± 0.011	
	Cu-Cu1	1.00 ± 0.5	2.955 ± 0.01	0.019 ± 0.002	
			PtCu		
	Cu-Cu1	3.28 ± 0.6	2.613 ± 0.01	0.019 ± 0.002	
	Cu-Pt1	5.34 ± 0.7	2.613 ± 0.01	0.019 ± 0.003	

	Scattering Path	*CN*	*R (Å)*	*σ^2(Å^2)*	*R-factor*
**Oxi. D_o**			***PtCu***		0.020
	Cu-Cu1	3.83 ± 0.6	2.644 ± 0.011	0.011 ± 0.001	
	Cu-Pt1	4.91 ± 1.0	2.644 ± 0.012	0.011 ± 0.002	
			Cu_2O		
	Cu-O1	0.24*	1.900 ± 0.070	0.004 ± 0.013	
	Cu-Cu1	0.73*	3.013 ± 0.011	0.011 ± 0.001	

	Scattering Path	*CN*	*R (Å)*	*σ^2(Å^2)*	*R-factor*
**Oxi. I_o**			***PtCu***		0.014
	Cu-Cu1	4.72 ± 1.7	2.641 ± 0.015	0.018 ± 0.005	
	Cu-Pt1	4.10 ± 1.1	2.647 ± 0.019	0.012 ± 0.003	
			Cu_2O		
	Cu-O1	0.10 ± 0.2	1.831 ± 0.010	0.003 ± 0.002	
	Cu-Cu1	0.16 ± 0.5	2.986 ± 0.015	0.018 ± 0.005	

	Scattering Path	*CN*	*R (Å)*	*σ²(Å²)*	*R-factor*
**Oxi. A_0**			**<u>*CuO*</u>**		0.008
	Cu-O1	1.19 ± 0.1	1.953 ± 0.04	0.009 ± 0.002	
	Cu-Cu1	2.39 ± 0.2	2.643 ± 0.002	0.017 ± 0.002	
	Cu-O2	1.59 ± 0.2	1.934 ± 0.04	0.009 ± 0.002	
			**<u>*Cu_2O*</u>**		
	Cu-O1	1.08 ± 0.4	1.993 ± 0.04	0.010 ± 0.002	
	Cu-Cu1	3.24 ± 0.1	2.900 ± 0.002	0.018 ± 0.002	

	Scattering Path	*CN*	*R (Å)*	*σ²(Å²)*	*R-factor*
**RT He**			**<u>*CuO*</u>**		0.011
	Cu-O1	2.90 ± 0.2	1.955 ± 0.008	0.004 ± 0.001	
	Cu-Cu1	5.71 ± 0.5	2.921 ± 0.02	0.020 ± 0.003	
	Cu-O2	3.81 ± 0.3	3.692 ± 0.02	0.004 ± 0.001	

*Parameters were fixed in the fitting and obtained as the product of N of the shell from the model and adjusted S_0^2 for the reference Cu.

REFERENCES

(1) Yin, M.; Wu, C.-K.; Lou, Y.; Burda, C.; Koberstein, J. T.; Zhu, Y.; O'Brien, S., Copper Oxide Nanocrystals. *J. Am. Chem. Soc.* **2005**, 127, 9506-9511.

(2) Christensen, G. L.; Langell, M. A., Characterization of Copper Palladium Oxide Solid Solutions by X-Ray Diffraction, X-Ray Photoelectron Spectroscopy, and Auger Electron Spectroscopy. *J. Phys. Chem. C* **2013**, 117, 7039-7049.

(3) Martini, A.; Alladio, E.; Borfecchia, E., Determining Cu–Speciation in the Cu–CHA Zeolite Catalyst: The Potential of Multivariate Curve Resolution Analysis of In Situ XAS Data. *Top. Catal.* **2018**, 61, 1396-1407.

(4) Staniuk, M.; Zindel, D.; Van Beek, W.; Hirsch, O.; Kränzlin, N.; Niederberger, M.; Koziej, D., Matching the Organic and Inorganic Counterparts During Nucleation and Growth of Copper-Based Nanoparticles - In Situ Spectroscopic Studies. *CrystEngComm* **2015**, 17, 6962-6971.
